# PDGFRα inhibition reduces myofibroblast expansion in the fibrotic rim and enhances recovery after ischemic stroke

**DOI:** 10.1172/JCI171077

**Published:** 2025-01-14

**Authors:** Jil Protzmann, Manuel Zeitelhofer, Christina Stefanitsch, Daniel Torrente, Milena Z. Adzemovic, Kirils Matjunins, Stella J.I. Randel, Sebastian A. Lewandowski, Lars Muhl, Ulf Eriksson, Ingrid Nilsson, Enming J. Su, Daniel A. Lawrence, Linda Fredriksson

**Affiliations:** 1 Department of Medical Biochemistry and Biophysics, Karolinska Institutet, Stockholm, Sweden.; 2 Department of Internal Medicine, University of Michigan Medical School, Ann Arbor, Michigan, USA.

**Keywords:** Neuroscience, Vascular biology, Fibrosis, Growth factors, Stroke

## Abstract

Ischemic stroke is a major cause of disability in adults. Early treatment with thrombolytics and/or thrombectomy can significantly improve outcomes; however, following these acute interventions, treatment is limited to rehabilitation therapies. Thus, identification of therapeutic strategies that can help restore brain function in the post-acute phase remains a major challenge. Here we report that genetic or pharmacologic inhibition of the PDGF-CC/PDGFRα pathway, which has previously been implicated in stroke pathology, significantly reduced myofibroblast expansion in the border of the fibrotic scar and improved outcome in a sensory-motor integration test after experimental ischemic stroke. This was supported by gene expression analyses of cerebrovascular fragments showing upregulation of profibrotic/proinflammatory genes, including genes of the TGF pathway, after ischemic stroke or intracerebroventricular injection of active PDGF-CC. Further, longitudinal intravital 2-photon imaging revealed that inhibition of PDGFRα dampened the biphasic pattern of stroke-induced vascular leakage and enhanced vascular perfusion in the ischemic lesion. Importantly, we found PDGFRα inhibition to be effective in enhancing functional recovery when initiated 24 hours after ischemic stroke. Our data implicate the PDGF-CC/PDGFRα pathway as a crucial mediator modulating post-stroke pathology and suggest a post-acute treatment opportunity for patients with ischemic stroke targeting myofibroblast expansion to foster long-term CNS repair.

## Abstract 2

This study suggests targeting myofibroblast expansion by inhibition of PDGFRα signaling as a potential novel post-acute target to foster CNS repair after ischemic stroke.

## Graphical abstract

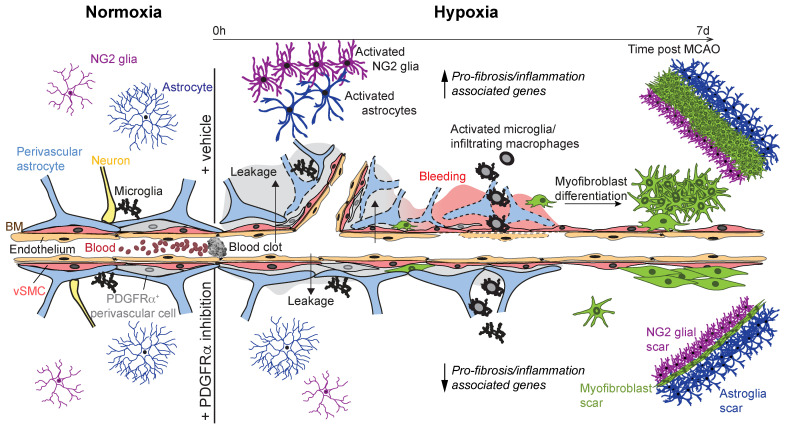

## Introduction

Ischemic stroke represents a major public health challenge and is currently the second leading cause of disability and death worldwide (1). Treatment options are limited to early treatment with intravenous thrombolysis with recombinant tissue plasminogen activator (tPA) and/or mechanical thrombectomy (2). Despite these acute interventions, up to 40% of patients will die or remain functionally dependent. More importantly, due to various contraindications, only a fraction of all patients with ischemic stroke will receive these therapies. Much effort has therefore been invested in researching additional treatment options, mainly focusing on direct neuroprotection. However, due to the limited success of neuroprotective approaches, studies examining the therapeutic potential of preserving or restoring the integrity of the blood-brain barrier (BBB) have been gaining interest (3).

Damage to the BBB is an early pathological event in ischemic stroke, and the absence of a functional BBB will lead to profound disturbances in neuronal and glial signaling (4). Several molecular pathways have been reported to play important roles in disease-induced BBB damage, including in our previous studies demonstrating a role for tPA-induced activation of PDGFRα signaling in perivascular cells in the neurovascular unit (NVU) via catalysis of the ligand PDGF-CC (5–11). Studies from our laboratory and others have shown that administering imatinib, an RTK inhibitor of PDGFRs, ABL, and c-KIT (12), significantly improves outcome after both ischemic and hemorrhagic stroke in rodents (9, 13, 14) and importantly in humans (15). The beneficial effect of imatinib has been ascribed to its ability to reduce stroke-induced BBB leakage, but how this exactly translates to improved neurological outcome is yet unknown.

It has previously been shown that cells in the NVU act as sensors for insults to the brain and that they elicit activation of the early response to reactive gliosis response, in which astrocytes, NG2-glia (also referred to as oligodendrocyte progenitor cells [OPCs]), and microglia become activated, leukocytes infiltrate, and a cascade of post-stroke neuropathology is initiated (16). The reactive gliosis response is an important early injury response (occurring hours to days after disease onset) that is considered to orchestrate the subsequent scar formation in the subacute/chronic phase after the insult (days to weeks after onset) (17). The CNS scar consists of an outer glial component formed by reactive glial cells and an inner fibrotic core harboring mainly fibroblasts and immune cells. Demarcation of the lesion by activated glia cells is thought to limit the spread of cellular death and confine chronic inflammation at the lesion site, thereby protecting the relatively unaffected surrounding CNS tissue. Scar formation is fundamental for injury resolution; however, it is also deleterious to functional recovery (18), as the glial component of the scar has been shown to have inhibitory effects on CNS axonal regrowth (19).

It has been proposed that poor CNS recovery after injury may be mechanistically similar to chronic/unresolved wounds in peripheral tissues (20). A key phase in the peripheral wound-healing process is the expansion (proliferation/migration/differentiation) of contractile myofibroblasts from yet-to-be-established progenitor cells (21). Myofibroblasts will contribute to repair by generating contractile forces enabling the surrounding tissue to contract and close the wound. Normally, after the successful completion of repair, the myofibroblast scar will resolve, but in cases of pathology, myofibroblasts accumulate and synthesize an excessive amount of extracellular matrix (ECM) with a composition different from that of normal tissue ECM (21). It has been shown that the increased stiffness and profibrotic composition of this ECM contributes to distortion of the parenchymal architecture, thus leading to compromised organ recovery and function. A master regulator of myofibroblast differentiation is TGF-β,which, through canonical signaling via SMAD mediators, will induce upregulation of characteristic myofibroblast markers, including α–smooth muscle actin (ASMA) and fibronectin (21). Interestingly, recent research has demonstrated functional crosstalk between the TGF-β and PDGFRα signaling pathways in regulating myofibroblast migration and differentiation in skeletal muscle regeneration (22), and PDGFRα has been implicated in the temporal control of fibroblast-to-myofibroblast transition in skin wound healing (23).

In the present study, we utilized a photothrombotic murine model of experimental ischemic stroke to investigate the interrelationship between stroke-induced cerebrovascular changes and CNS repair. Using longitudinal in vivo 2-photon microscopy (24), we found that imatinib blocked stroke-induced vascular leakage, which coincided with preserved cellular organization in the NVU during the acute phase after ischemic stroke. Gene expression analyses of cerebrovascular fragments isolated from the ipsilateral hemisphere of imatinib-treated mice and their vehicle-treated controls identified differential expression of pathways associated with inflammation and fibrosis. Immunohistological analyses showed that imatinib dampened the acute reactive gliosis response and subsequent myofibroblast expansion after stroke, while having very limited effect on the remainder of the glial scar. Assessment of a sensory-motor integration test revealed that functional benefit with imatinib treatment progressively improved over time and, importantly, also demonstrated the efficacy of imatinib treatment when administered in the post-acute phase after ischemic onset. Using an anti–PDGF-CC neutralizing antibody (25, 26) and genetic ablation of *Pdgfra* in glial fibrillary acidic protein–positive (GFAP^+^) cells, we were able to confirm that the effect of imatinib on myofibroblast expansion was mediated via activation of the PDGF-CC/PDGFRα pathway. Taken together, our results suggest myofibroblast expansion as a potential post-acute target to foster CNS repair after ischemic stroke.

## Results

### Imatinib preserves BBB integrity and organization of the NVU during the acute phase after middle cerebral artery occlusion.

**Figure 1:**
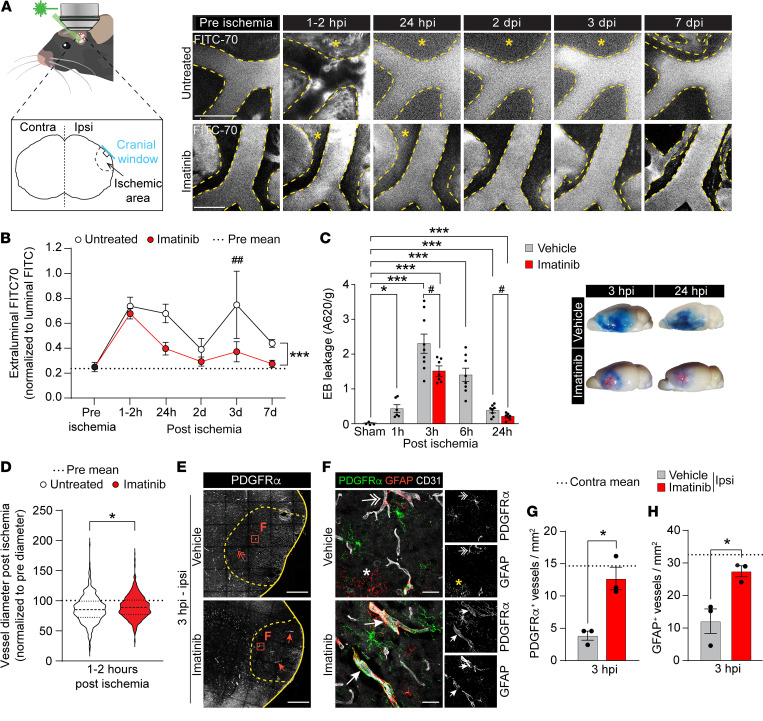
Imatinib attenuates MCAO-induced cerebrovascular breach and vasoconstriction. (**A**) Two-photon images of FITC70 signal before ischemia (pre) and at different time points after ischemia. Asterisks: extraluminal FITC70 signal. (**B**) Quantification of 2-photon extraluminal FITC70 signal (*n* = 4). *P < 0.05, treatment effect; *^#^P* < 0.05 relative to control at that time point. Pre mean indicates mean value prior to ischemia. (**C**) Analysis and representative images of EB extravasation in the ipsilateral ischemic hemisphere in the acute phase after MCAO (*n* = 4–9). *P < 0.05, relative to sham; *^#^P* < 0.05, relative to vehicle controls. (**D**) Quantification of relative vessel diameter change 1–2 hpi compared with the diameter before onset. Recorded with longitudinal 2-photon microscopy in endothelial reporter mouse vessels (*n* = 399 untreated; 609 imatinib-treated) from 4 animals per treatment. (**E** and **F**) Ipsilateral overviews (**E**) and high-magnification images from the ischemic area (**F**) of immunofluorescence staining for PDGFRα and GFAP in brain sections collected at 3 hpi. Vessels were visualized with CD31. Arrows: perivascular expression of PDGFRα and GFAP; 2-headed arrows, scattered/lost perivascular expression of PDGFRα and GFAP; asterisk, non-perivascular GFAP signal. Ischemic area outlined with dashed lines. (**G** and **H**) Quantification of PDGFRα^+^ (**G**) and GFAP^+^ (**H**) vessels (*n* = 3). contra, contralateral; ipsi, ipsilateral. Representative images of maximum-intensity projections (**A** and **F**) and single-plane images (**E**) from vehicle- and imatinib-pretreated mice. Data points represent individual animals; bars, group mean ± SEM (**C**, **G**, and **H**); in **B**, data points represent group mean ± SEM. The dashed lines in **B**, **D**, **G**, and **H** show the pre-ischemia/contralateral group mean. Mixed-effects analysis with Tukey’s post-hoc test (**B**); 1-way ANOVA with Welch’s test (**C**); 2-tailed, unpaired *t* test with Welch’s correction (**D**, **G**, and **H**). *P < 0.05, ^#^*P* < 0.05; **P < 0.01; ***P < 0.001; ^##^*P* < 0.01. Scale bars: 100 μm (**A**); 500 μm (**E**); 25 μm (**F**).

TPA-mediated activation of PDGF-CC/PDGFRα signaling in the NVU during ischemic stroke in mice induces opening of the BBB and augments brain injury (9). Blocking this pathway with imatinib, an RTK inhibitor, improves outcome following ischemic stroke in both mice (9) and humans (15). To further delineate the downstream mechanism of PDGF-CC/PDGFRα signaling in ischemic stroke, we performed vascular leakage, gene expression, and immunofluorescence analyses in mice treated with imatinib or anti–PDGF-CC neutralizing antibody (pretreatment if not stated otherwise); or in mice in which perivascular PDGFRα had been ablated (see study outline in Supplemental Figure 1A; supplemental material available online with this article; https://doi.org/10.1172/JCI171077DS1). Utilizing intravital 2-photon imaging of stroke-induced BBB breach (24), which enables longitudinal studies of vascular leakage following middle cerebral artery occlusion (MCAO) of the cortical segment of the MCA, we determined the kinetics of extraluminal cerebral leakage from the first hours after ischemia (hours post-ischemia [hpi]) to 7 days after ischemia (days post-ischemia [dpi]) (asterisks, Figure 1A). We found that imatinib significantly reduced ischemic stroke–induced extravasation of intravenously administered fluorescent dye FITC70 into the brain parenchyma compared with control treatment (Figure 1B). In accordance with previously published data (27), we observed a biphasic pattern of vascular leakage, with the first peak of extraluminal FITC70 occurring within hours after ischemia and the second at 3 dpi. We found that both waves were reduced by imatinib treatment. Further analysis of the first wave of MCAO-induced BBB leakage with Evans blue (EB) dye revealed that at1 hpi, the BBB was already losing its integrity (Figure 1C). At 3 hpi we detected the highest level of leakage, which was followed by a time-dependent decrease in EB extravasation. Imatinib treatment significantly reduced MCAO-induced EB extravasation at both 3 and 24 hpi compared with vehicle treatment (Figure 1C). This coincided with a significantly lower number of microbleeds at 3 hpi, as detected by immunofluorescence staining for the red blood cell marker TER119, mainly around medium- to large-diameter vessels in the ischemic area in imatinib-treated mice compared with vehicle-treated controls (Supplemental Figure 1, B–D).

It is plausible that the MCAO-increased permeability was due to a loss of endothelial tight junctions (TJs), and we therefore performed staining with the TJ marker claudin-5 (CLDN5) at 3 hpi, a time point when extensive BBB breach was observed. Our analysis revealed no significant difference between the treatment groups in CLDN5 immunofluorescent signal in vessels in the ischemic area at 3 hpi (Supplemental Figure 1, E–H), which is in line with the current literature suggesting that the first wave of BBB breakdown in ischemic stroke is driven by increased transendothelial transport and not the result of increased paracellular permeability due to TJ disassembly (28, 29). The possibility should be noted, however, that TJ modifications such as phosphorylation — as shown for, e.g., occludin (30) — might affect BBB integrity despite normal expression and localization of TJ markers.

Our staining for the endothelial marker CD31 revealed that MCAO provoked a decrease in the number of vessels with a diameter greater than 10 μm in the ischemic region of vehicle controls at 3 hpi and that this was significantly alleviated by imatinib treatment (Supplemental Figure 1I). It is possible that these MCAO-provoked vascular changes were caused by vascular constriction, vascular rarefaction, collateral recruitment, etc., and to test this we performed 2-photon analysis of vessel diameter in endothelial cell reporter mice. This revealed a global vessel constriction (compared with vessel diameter before ischemia) affecting all vessel types in the vascular tree 1–2 hpi (Figure 1D). Assessment of the change in vessel diameter along the arteriovenous axis showed that the arterial segments in both untreated and imatinib-treated mice were the most constricted. Imatinib significantly mitigated vasoconstriction in the arterial and venous segments, while capillary diameter was unchanged (Supplemental Figure 1, J–L). Further analyses of the NVU demonstrated that imatinib treatment preserved normal levels of perivascular expression of PDGFRα as well as perivascular expression of GFAP around medium- to large-sized vessels within the ischemic area at 3 hpi (arrows, Figure 1, E–H). In contrast, the immunoreactivity of these 2 markers was largely lost or scattered around vessels in the ischemic area of vehicle controls (2-headed arrows), whereas nonvascular GFAP signal appeared to be increased (asterisks, Figure 1F). Taken together, these data suggest that imatinib treatment maintains vascular health after ischemic stroke.

### Imatinib regulates expression of genes associated with fibrosis and inflammation in the cerebrovasculature after MCAO.

**Figure 2:**
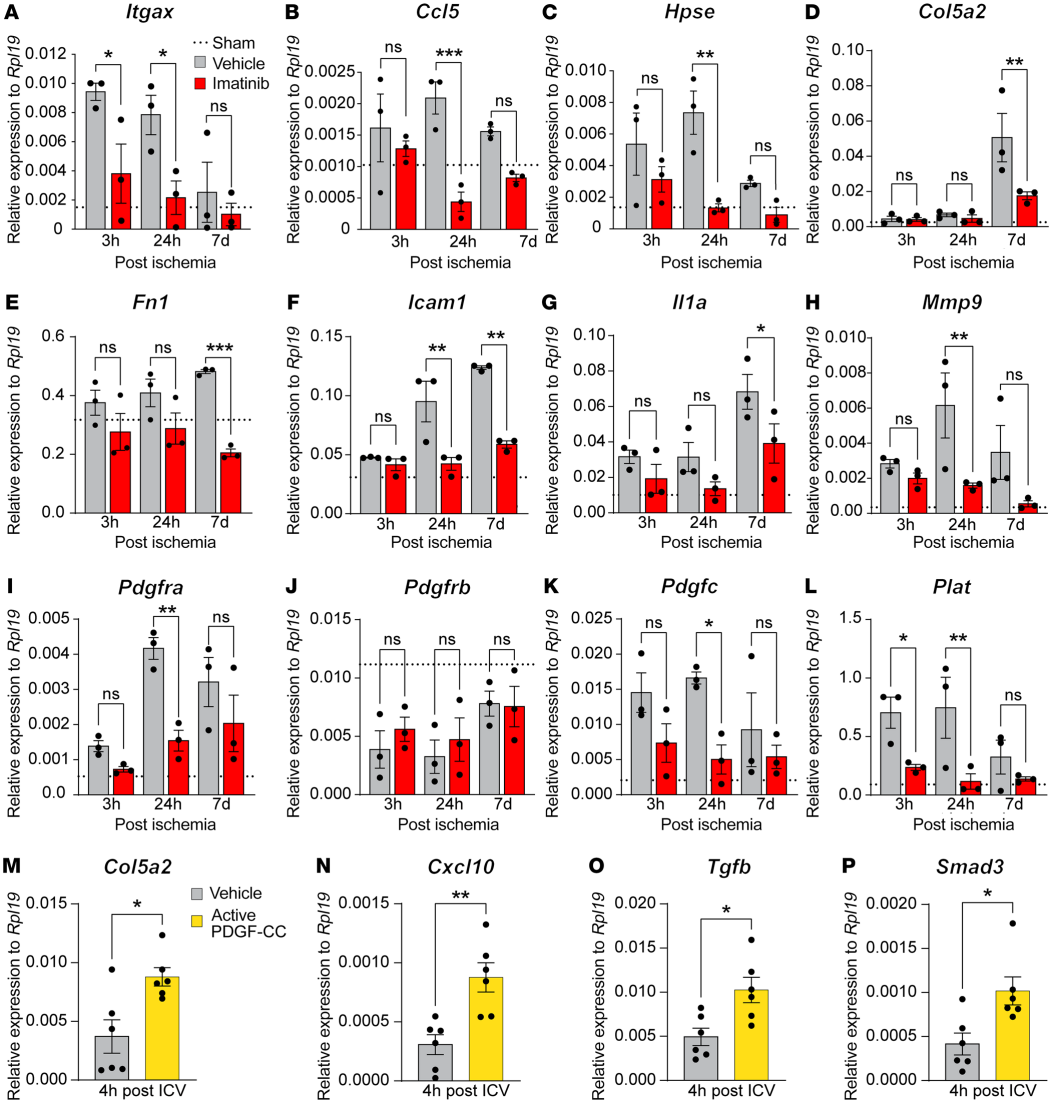
Imatinib dampens MCAO-induced expression, and PDGF-CC provokes expression, of profibrotic/proinflammatory genes in the cerebrovasculature. Gene expression analysis on RNA isolated from cerebrovascular fragments collected from the ipsilateral hemisphere of vehicle- and imatinib-pretreated mice at different time points after MCAO (**A**–**L**) or 4 hours after ICV injection with active PDGF-CC protein in naive mice (**M**–**P**). (**A**–**L**) qPCR analysis of differentially expressed genes in the ischemic cerebrovasculature of vehicle- and imatinib-pretreated mice (*n* = 3). (**M**–**P**) qPCR analysis of expression of common fibrotic genes in cerebrovascular fragments isolated from WT mice 4 hours after ICV injection of either vehicle or active PDGF-CC protein (*n* = 6). Data points represent individual animals; dashed lines show the mean for the sham-operated group. Two-way ANOVA with uncorrected Fisher’s least significant difference test (**A**–**L**); 2-tailed, unpaired *t* test with Welch’s correction (**M**–**P**). ns, nonsignificant; *P < 0.05; **P < 0.01; ***P < 0.001.

Based on these findings, we isolated cerebrovascular fragments at different time points after MCAO from mice treated with imatinib or their vehicle-treated controls, and performed gene expression and pathway analyses. Assessment of gene expression in the cerebrovasculature identified 121 and 85 differentially expressed transcripts at 3 and 24 hpi, respectively (Supplemental Figure 2A and Supplemental Tables 1 and 2), of which some of the top regulated genes were validated by quantitative PCR (qPCR) analysis (Figure 2, A–C, and Supplemental Figure 2, B and C). Pathway analysis revealed that functions related to fibrosis, vascular damage, inflammation, as well as leukocyte adhesion were modulated by imatinib treatment (Supplemental Figure 2, D and E). Subsequent comparison to the Harmonizome database (31) confirmed these findings and revealed that approximately 30% of the imatinib-regulated transcripts were associated with fibrosis and 15% with inflammation at both 3 and 24 hpi (Supplemental Figure 2, F and G). Since imatinib has shown benefit in experimental models of hemorrhagic stroke (13) and reduced the number of microbleeds (Supplemental Figure 1D), we compared our microarray data with the publicly available microarray dataset from the perihematomal area of patients with stroke (GEO GSE24265) (32), an area associated with BBB disruption and high levels of edema formation (33). The analysis revealed a high level of overlap, and approximately half of the overlapping genes were found to be associated with fibrosis (Supplemental Figure 2, H and I, and Supplemental Tables 3 and 4), thus suggesting that the results of our murine analyses might be of relevance for human stroke pathology.

As the highest proportion of differentially regulated genes after imatinib treatment was associated with fibrosis and inflammation, we performed qPCR analysis on cerebrovascular fragments isolated at 3 hpi, 24 hpi, and 7 dpi to analyze expression of selected profibrotic/proinflammatory genes. We found that MCAO-induced expression of the fibrosis-associated genes *Itgax*, *Ccl5*, *Hpse*, *Col5a2*, *Fn1*, *Icam1*, *Il1a*, and *Mmp9* was significantly reduced at various time points by imatinib treatment (Figure 2, A–H). Many of these genes, e.g., *Ccl5*, *Icam1*, *Il1a*, and *Itgax*, have also been associated with the inflammatory response. We further investigated the effect of imatinib treatment on the expression of genes within the PDGF-CC/PDGFRα pathway by qPCR. In accordance with our transcriptome analysis, MCAO-induced cerebrovascular expression of *Pdgfra* was significantly inhibited in imatinib- compared with vehicle-treated animals at 24 hpi (Figure 2I), while gene expression of *Pdgfrb* was unaffected by imatinib treatment (Figure 2J). In addition, both *Pdgfc* and *Plat* (the gene encoding for tPA) were downregulated by imatinib compared with vehicle control treatment (Figure 2, K and L).

To establish whether the PDGF-CC/PDGFRα pathway can trigger expression of fibrotic genes in the cerebrovasculature per se, we administered active PDGF-CC protein by intracerebroventricular (ICV) injection in naive mice and isolated cerebrovascular fragments 4 hours later. qPCR analysis revealed that PDGF-CC injection led to significant cerebrovascular upregulation of the fibrosis- and inflammation-associated genes *Col5a2* and *Cxcl10* (Figure 2, M and N). Both these genes were found to be downregulated following imatinib treatment after MCAO (Figure 2D and Supplemental Table 1). Interestingly, members of the TGF-β signaling pathway (*Tgfb* and *Smad3*), known master regulators of myofibroblast differentiation (21) and key to epithelial/endothelial-mesenchymal transition (EMT/EndoMT) (34), were significantly upregulated after ICV injection of active PDGF-CC (Figure 2, O and P).

### Imatinib attenuates the reactive gliosis response within hours after MCAO.

**Figure 3:**
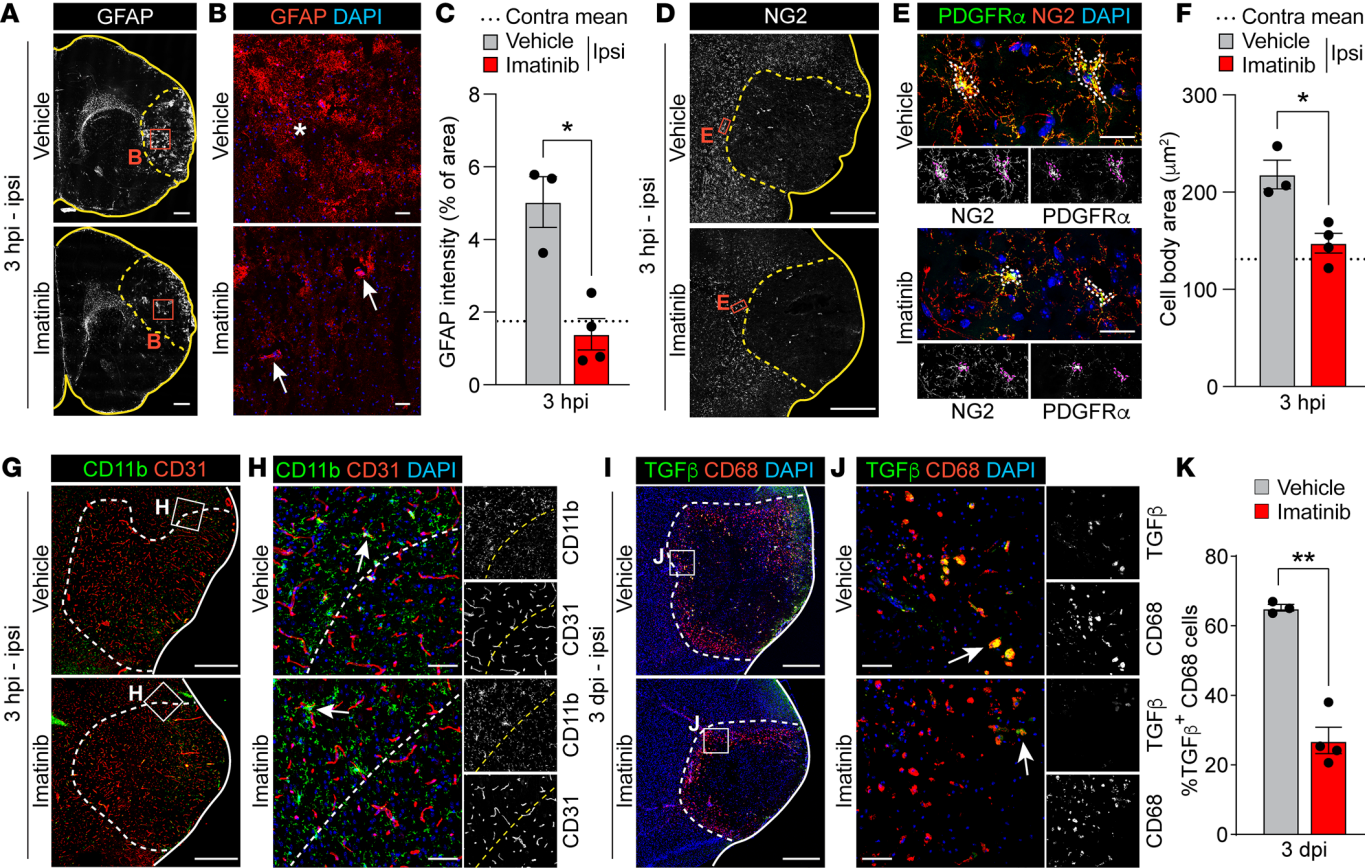
Imatinib attenuates the reactive gliosis response after MCAO. (**A** and **B**) Ipsilateral overviews (**A**) and high-magnification images from the ischemic area (**B**) of staining for GFAP. Asterisk: nonperivascular GFAP signal; arrows: perivascular GFAP signal. (**C**) Quantification of GFAP expression based on antibody immunoreactivity intensity above a set threshold (*n* = 3–4). (**D** and **E**) Ipsilateral overviews (**D**) and high-magnification images from the ischemic border (**E**) of costaining for NG2 and PDGFRα. Double-positive cell bodies outlined in **E**. (**F**) Quantification of PDGFRα^+^NG2^+^-glia cell size in the peri-ischemic area (outlined in **E**) (*n* = 3–4). (**G** and **H**) Ipsilateral overviews (**G**) and high-magnification images from the ischemic border (**H**) of staining for CD11b. Vessels visualized with CD31. Arrows: condensed CD11b^+^ microglia/infiltrating macrophages. (**I** and **J**) Ipsilateral overviews (**I**) and high-magnification images from the ischemic border (**J**) of costaining for CD68 and TGF-β. (**K**) Quantification of TGF-β–expressing CD68^+^ microglia/infiltrating macrophages (arrows in **J**) in the ischemic area (*n* = 3–4). Representative images of immunofluorescence staining and quantification in brain sections from vehicle- and imatinib-pretreated mice collected at 3 hpi (**A**–**H**) or 3 dpi (**I**–**K**). Stitched epifluorescence images (**A**), single-plane confocal images (**B**, **G**, and **I**), and maximum-intensity projections of confocal *Z*-stacks (**D**, **E**, **H**, and **J**). Ischemic area outlined with dashed lines. Data points represent individual animals; bars, group mean ± SEM; and dashed line, contralateral group mean. Two-tailed, unpaired *t* test with Welch’s correction (**C**, **F**, and **K**). *P < 0.05; **P < 0.01. Scale bars: 500 μm (**A**, **D**, **G**, and **I**); 50 μm (**B**, **H**, and **J**); 25 μm (**E**).

Given the above findings implicating PDGF-CC/PDGFRα in the fibrotic injury response, we next investigated the effect of imatinib on reactive gliosis, the immediate early injury response in the CNS known to orchestrate the formation of the fibrotic scar (17). Among the glial cells taking part in reactive gliosis are astrocytes, NG2-glia, and microglia, which are activated/recruited to the site of insult. Since astrocytes are known to react to injury by hypertrophy and upregulation of GFAP in the acute phase following the insult (35), we assessed astrogliosis by staining for GFAP at 3 hpi. We found increased nonvascular GFAP signal (asterisks) in the ischemic area compared with the nonischemic surrounding tissue in vehicle-treated controls (Figure 3, A and B), which was significantly reduced in imatinib-pretreated animals (Figure 3C). The difference in nonvascular GFAP signal between treatment groups at 3 hpi was not due to a difference in the total number of astrocytes, as staining for the astrocyte-specific nuclear marker SOX9 detected no difference in the number of SOX9^+^ nuclei in the ischemic area between vehicle- and imatinib-treated animals (Supplemental Figure 3, A–C). Further analysis of the reactive gliosis response showed that NG2-glia cell body condensation, determined by staining for neuron glia antigen-2/CSPG4 (NG2) and PDGFRα, commenced in the ischemic border of vehicle control brains at 3 hpi (Figure 3, D and E) and was significantly inhibited by imatinib treatment (Figure 3F). The majority of these PDGFRα^+^ NG2-glia expressed OLIG2, suggesting they were OPCs (Supplemental Figure 3, D and E). Since OPCs are progenitors of myelinating oligodendrocytes and our transcriptome analyses identified myelin basic protein (*Mbp*) as an imatinib-regulated gene (Supplemental Tables 1 and 2, demonstrating downregulation at 3 hpi and upregulation at 24 hpi following imatinib treatment), we assessed expression of MBP in brain sections 3 hpi. These analyses, however, did not detect any difference in myelination between imatinib-treated and control mice at 3 hpi (Supplemental Figure 3, F and G).

The reactive gliosis response also includes activation/recruitment of microglia/macrophages to the lesion site. Staining for CD11b, a marker for microglia/infiltrating macrophages, however, did not show any difference in microgliosis/infiltrating macrophages at 3 hpi, and condensation of microglia was apparent in the ischemic border (arrows) of both vehicle- and imatinib-treated mice (Figure 3, G and H). The condensed CD11b^+^ microglia coexpressed IBA1, another microglia/macrophage marker, but were negative for PDGFRα (Supplemental Figure 3H). In our analyses, the condensed CD11b^+^IBA1^+^ microglia were often found in close proximity to PDGFRα^+^ cells (arrows, Supplemental Figure 3I), suggesting efficient activation of PDGF-CC/PDGFRα signaling, which has been shown to be dependent on Mac-1 on microglia (36). Further analysis of the microglia/macrophage response to MCAO and imatinib treatment revealed, in line with recent findings in rhesus monkeys (37), a high number of CD68^+^ activated microglia/macrophages in the ischemic border at 3 dpi, of which approximately 60% stained positive for the profibrotic marker TGF-β in vehicle-treated animals (Figure 3, I–K). Imatinib treatment significantly reduced the number of TGF-β–expressing CD68^+^ cells (Figure 3K), which is particularly interesting considering our data showing that PDGF-CC signaling regulated expression of genes in the TGF pathway (Figure 2, O and P). In addition, we found that imatinib significantly reduced the number of CD11b^+^ ameboid microglia/infiltrating macrophages in the ischemic core at 7 dpi compared with control treatment (Supplemental Figure 3, J–L), while displaying seemingly limited effect on peripheral immune cell infiltration (neutrophil, B cell, and T cell infiltration) (Supplemental Figure 4). Taken together, these results indicate that imatinib specifically attenuates the reactive gliosis response induced by MCAO, with major effects seen on dampening activation of astrocytes, NG2-glia, and microglia/macrophages.

### Imatinib specifically targets myofibroblast expansion in the rim of the fibrotic scar.

**Figure 4:**
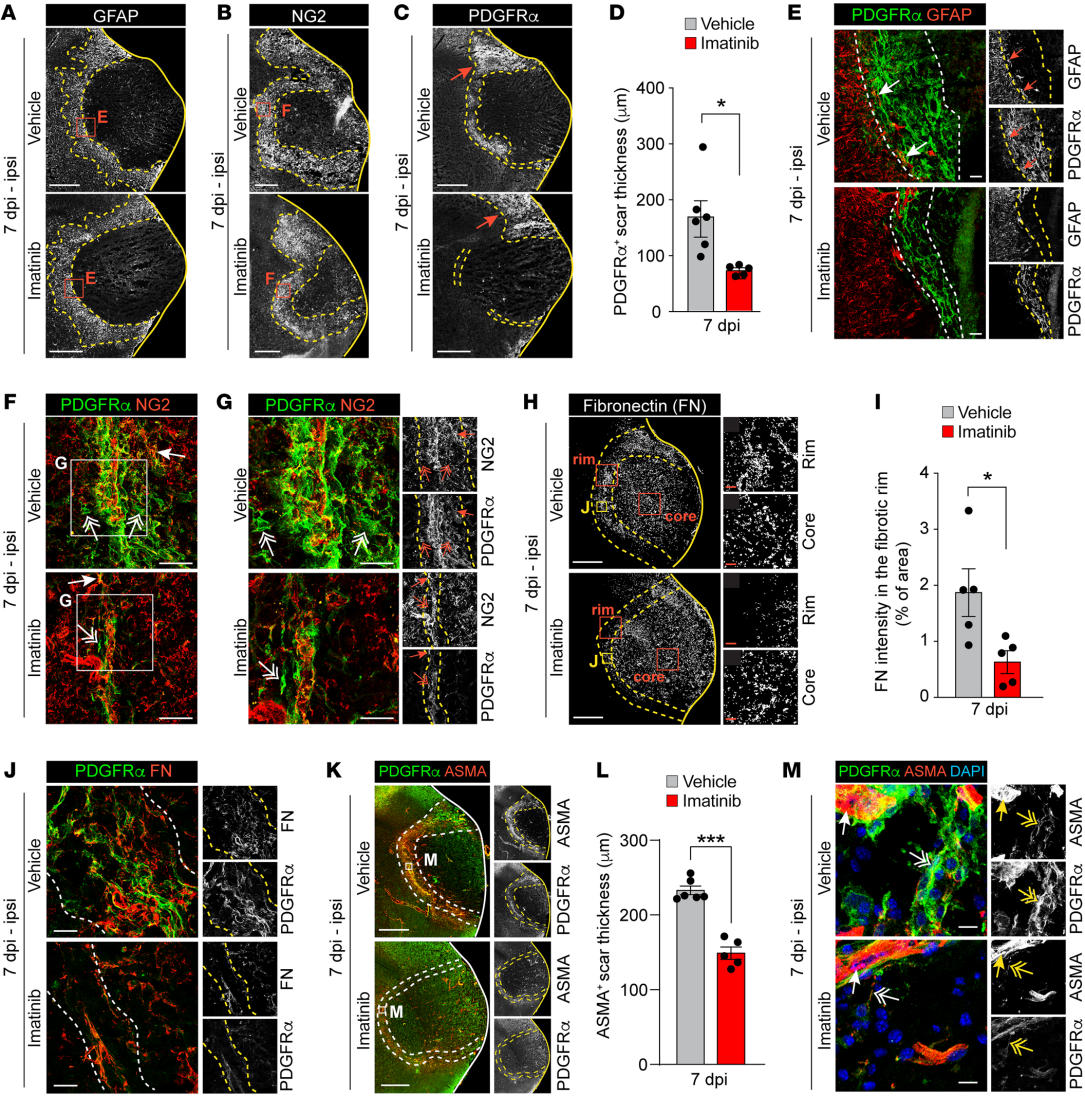
Imatinib specifically targets expansion of a PDGFRα^+^ myofibroblast scar in the fibrotic rim after MCAO. Representative images of immunofluorescence staining and quantification in brain sections from vehicle- and imatinib-pretreated mice collected at 7 dpi. (**A**–**C**) Ipsilateral overviews from staining for GFAP (**A**), NG2 (**B**), and PDGFRα (**C**). Arrows in **C**: PDGFRα^+^ scar not targeted by imatinib. (**D**) Quantification of PDGFRα^+^ scar thickness in the fibrotic rim (demarcated in **C**) (*n* = 5–6). (**E**) High-magnification images from the fibrotic rim from costaining for PDGFRα and GFAP. Arrows: PDGFRα^+^GFAP^+^ cells. (**F** and **G**) Costaining for PDGFRα and NG2 acquired within the NG2^+^ glial scar. Arrows: PDGFRα^+^NG2^+^ cells; 2-headed arrows: non-perivascular PDGFRα^+^NG2^–^ cells. (**H**) Ipsilateral overview and magnifications of the fibrotic rim and core from staining for fibronectin (FN). (**I**) Quantification of FN expression in the fibrotic rim (demarcated in **H**) (*n* = 5). (**J**) High-magnification images from the fibrotic rim of costaining for PDGFRα and fibronectin. (**K**) Ipsilateral overviews from staining for ASMA and PDGFRα. (**L**) Quantification of ASMA^+^ scar thickness in the fibrotic rim (demarcated in **K**) (*n* = 5–6). (**M**) High-magnification images from the fibrotic rim of costaining for ASMA and PDGFRα. Two-headed arrows: PDGFRα^+^ASMA^+^ nonvascular cells; arrows: ASMA^+^ vSMCs. Stitched epifluorescence images (**A**–**C** and **K**), single-plane confocal images (**E** and **H**), and maximum-intensity projections of confocal *Z*-stacks (**F**, **G**, **J**, and **M**). Dashed lines demarcate glial scar (**A** and **B**) and myofibroblast scar (**C**–**K**). Data points represent individual animals; bars, group mean ± SEM. Two-tailed, unpaired *t* test with Welch’s correction (**D**, **I**, and **L**). *P < 0.05; ***P < 0.001. Scale bars: 500 μm (**A**–**C**, **H**, and **K**); 50 μm (**E** and **F**, core/rim in **H**); 25 μm (**G** and **J**); 10 μm (**M**).

Since reactive gliosis has been suggested to orchestrate the subsequent formation of the glial and fibrotic scar (17), we next investigated whether the early effect of imatinib on reactive gliosis affected scar formation in the early chronic tissue remodeling phase at 7 dpi. Immunofluorescence staining for the astroglial scar with GFAP (Figure 4A and Supplemental Figure 5A) and the NG2-glial scar (Figure 4B and Supplemental Figure 5B) revealed that imatinib treatment did not markedly affect glial scar formation. However, we found that, compared with vehicle control, imatinib significantly reduced the formation and organization of a PDGFRα^+^ scar (Figure 4, C–G), along the rim of a collagen I–rich (COL1-rich) fibrotic scar (Supplemental Figure 5, C–E). Our analyses revealed that the PDGFRα+ scar was adjacent to the GFAP^+^ scar, with some overlapping expression right at the border (arrows, Figure 4E). Further, we found that the PDGFRα^+^ scar was embedded within the NG2^+^ scar, although the majority of the parenchymal PDGFRα^+^ cells in the fibrotic rim were negative for NG2 (2-headed arrows, Figure 4, F and G) and OLIG2 (Supplemental Figure 5, F and G). We noted that the OLIG2^+^ cells accumulated on the astroglial side of the scar, and we detected no difference between imatinib- and vehicle-treated mice. This was supported by staining for MBP, which revealed no difference in MBP immunoreactivity between the 2 treatment groups (Supplemental Figure 5H).

To further characterize the fibrotic scar, we performed immunofluorescence staining for the ECM glycoprotein fibronectin, a canonical myofibroblast gene (21). In line with the results from the gene expression analysis (Figure 2E), we found that imatinib reduced deposition of fibronectin (Figure 4H); this occurred selectively in the highly nucleated fibrotic rim without affecting expression in the lesion core (Figure 4I and Supplemental Figure 5I). Costaining for fibronectin and PDGFRα revealed that the PDGFRα^+^ cells in the fibrotic rim were embedded within the fibronectin-positive ECM, suggesting that fibronectin is secreted by these cells (Figure 4J), thus indicating a myofibroblast identity for the PDGFRα^+^ cells. Since parenchymal de novo expression of ASMA is a hallmark of myofibroblasts, we investigated the expression of ASMA in the PDGFRα^+^ fibrotic border at 7 dpi. We found that MCAO induced pronounced ectopic expression of ASMA in the PDGFRα^+^ fibrotic rim of vehicle controls, which was significantly reduced by imatinib treatment (Figure 4, J–M, and Supplemental Figure 5, J and K). Higher-magnification images revealed that ASMA was coexpressed in these PDGFRα^+^ cells, indicating they were indeed myofibroblasts (2-headed arrows), and we detected very few myofibroblasts in imatinib-treated animals (Figure 4M). In contrast, ASMA expression in vascular smooth muscle cells (vSMCs) appeared normal in both treatment groups, and vSMCs did not coexpress PDGFRα (arrows, Figure 4M; single confocal plane, Supplemental Figure 5K). It should be noted that in the unchallenged naive brain, ASMA expression was restricted to vSMCs surrounding medium- to large-sized vessels (arrow), and no parenchymal (nonvascular) expression was detected (asterisk, Supplemental Figure 5L). Staining with the proliferation marker Ki-67 showed high MCAO-induced proliferation within the fibrotic rim 7 dpi, which was nonsignificantly reduced following imatinib treatment (Supplemental Figure 5, M–O). Of note, our analyses showed that it was not only PDGFRα^+^ myofibroblasts that proliferated in the fibrotic rim (as assessed from costaining of Ki-67 and PDGFRα; arrowheads, Supplemental Figure 5N). Taken together, these data suggest that imatinib selectively inhibits myofibroblast expansion in the fibrotic rim after MCAO.

### PDGFRα drives myofibroblast expansion in the fibrotic rim.

**Figure 5:**
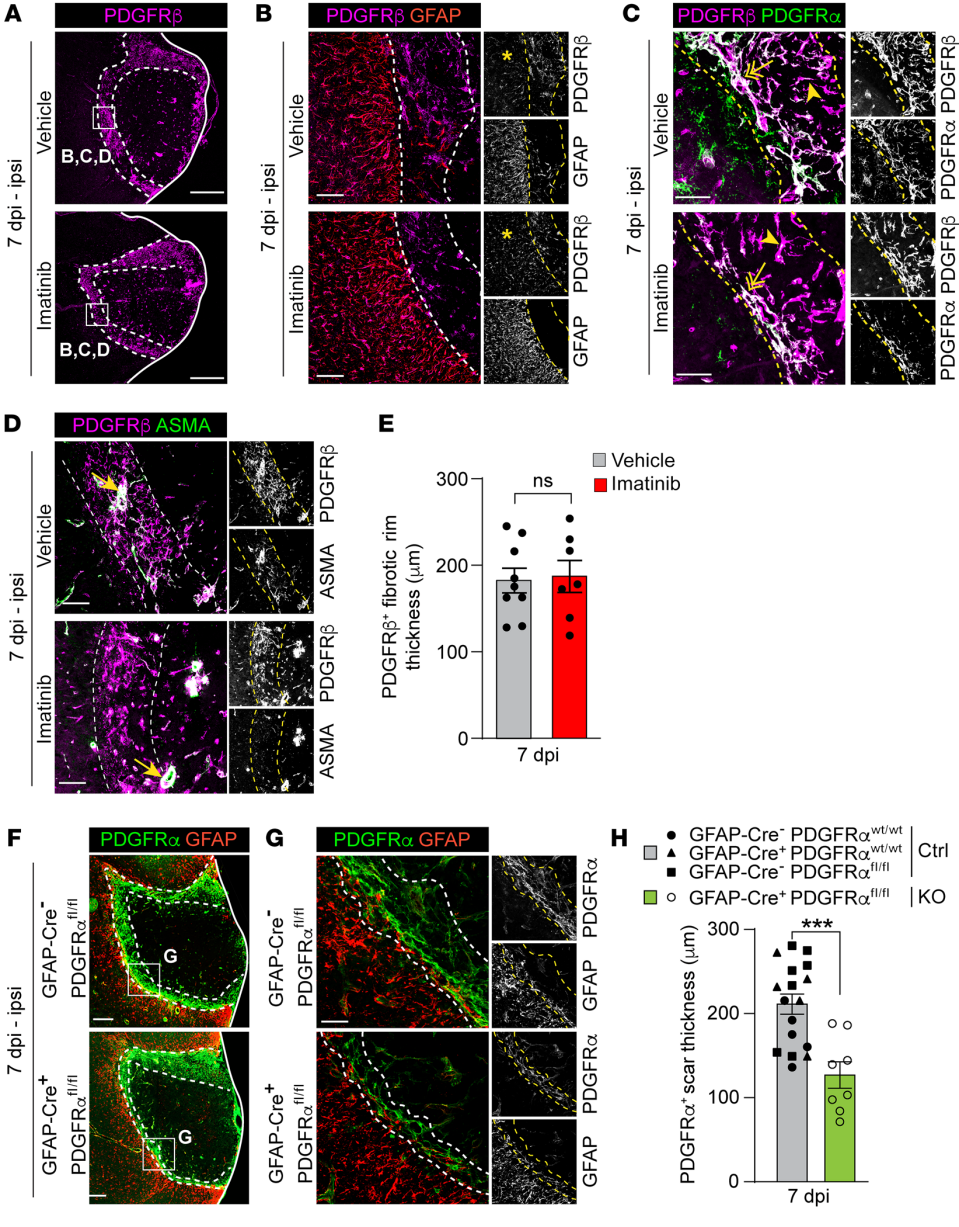
PDGFRα signaling drives expansion of the myofibroblast scar after MCAO. Representative images of immunofluorescent stainings and quantifications in brain sections from vehicle and imatinib pretreated mice (**A**–**E**) and GFAP-Cre;PDGFRα floxed mice (**F**–**H**) collected at 7 dpi. (**A**) Ipsilateral overview from staining for PDGFRβ. (**B**) High-magnification images from the fibrotic rim of costaining for PDGFRβ and GFAP. Asterisks: PDGFRβ expression within the astroglial scar. (**C**) High-magnification images from the fibrotic rim of PDGFRα and PDGFRβ costaining. Two-headed arrows: PDGFRα^hi^PDGFRβ^hi^ cells; arrowheads; PDGFRα^lo^PDGFRβ^hi^ cells. (**D**) High-magnification images from the fibrotic rim of costaining for PDGFRβ and ASMA. Arrows: ASMA^+^PDGFRβ^+^ vSMCs. (**E**) Quantification of PDGFRβ^+^ scar thickness in the fibrotic rim (demarcated in **A**) (*n* = 7–9). Ipsilateral overviews (**F**) and high-magnification images from the fibrotic rim (**G**) of staining for PDGFRα and GFAP in GFAP-Cre;PDGFRα floxed mice. (**H**) Quantification of PDGFRα^+^ scar thickness in the fibrotic rim (demarcated in **F**). *n* = 17 controls (ctrl), *n* = 8 PDGFRα knockouts (KO). Single-plane (**A**) and maximum-intensity projections (**B**–**D** and **G**) of confocal images, and stitched epifluorescent tiles (**F**). Dashed lines demarcate the PDGFRβ^+^ dense scar (**A**–**D**) and the myofibroblast scar (**F** and **G**) in the fibrotic rim. Data points represent individual animals and bars the group mean ± SEM. Two-tailed, unpaired *t* test with Welch’s correction (**E** and **H**). ***P < 0.001. Scale bars: 500 μm (**A** and **F**); 100 μm (**B** and **D**); 50 μm (**C** and **G**).

Since imatinib inhibits signaling not only via PDGFRα but also via PDGFRβ, we investigated the effect of imatinib on PDGFRβ, which is abundantly expressed in the fibrotic scar after CNS injury (38, 39). Analysis of PDGFRβ expression in the lesion at 7 dpi showed PDGFRβ immunoreactivity throughout the lesion, with the highest signal detected in the fibrotic rim, adjacent to the GFAP^+^ astroglia border (Figure 5, A and B, and Supplemental Figure 6A). Although, contrary to what we found with the PDGFRα^+^ scar, the margin between the astroglial scar and the PDGFRβ^+^ scar was not as distinct, with many activated astrocytes within the astroglia scar displaying de novo expression of PDGFRβ (asterisks, Figure 5B). Costaining for PDGFRβ with PDGFRα and/or ASMA revealed that ASMA^+^PDGFRα^+^ myofibroblasts coexpressed PDGFRβ (Figure 5, C and D, and Supplemental Figure 6B). In fact, we found that in vehicle controls, the vast majority of PDGFRβ^+^ cells coexpressed PDGFRα and ASMA in the fibrotic rim, which would suggest not only that all myofibroblasts were PDGFRβ^+^, but also that (nearly) all PDGFRβ^+^ cells in the fibrotic rim were myofibroblasts. Surprisingly, our analyses revealed that the thickness of the PDGFRβ^+^ scar in the fibrotic rim was unaffected by imatinib treatment (Figure 5E), even though the PDGFRα^+^ASMA^+^ scar was reduced (Figure 5, C and D). Further analyses of the myofibroblast scar demonstrated a population of PDGFRα^+^PDGFRβ^+^ cells within the fibrotic rim adjacent to the GFAP^+^ astroglial scar with high expression of both receptors (referred to as PDGFRα^hi^PDGFRβ^hi^; 2-headed arrows, Figure 5C), while PDGFRα^+^PDGFRβ^+^ cells located within the fibrotic rim but toward the fibrotic core exhibited lower expression of PDGFRα (referred to as PDGFRα^lo^PDGFRβ^hi^; arrowheads; Figure 5C). Taken together, our data suggest the existence of subpopulations of myofibroblasts within the fibrotic rim, possibly originating from different progenitor cells.

Based on the collective findings reported above, and the published data suggesting perivascular cells as the progenitors of myofibroblasts (40), we speculated that PDGFRα signaling in the NVU might be driving the expansion of the myofibroblast scar. To test this, we utilized GFAP-Cre;PDGFRα floxed mice, in which PDGFRα is genetically ablated from GFAP-expressing cells. We utilized this mouse line since in the nonischemic murine brain, PDGFRα expression is detected in GFAP^+^PDGFRβ^+^ perivascular cells around medium- to large-sized vessels (arrows, Supplemental Figure 6, C–E). GFAP-Cre^+^;PDGFRα^fl/fl^ mice have been shown to display a greater than 60% reduction in perivascular PDGFRα signal in the murine brain compared with littermate controls (7). We found that loss of perivascular PDGFRα in GFAP-Cre^+^;PDGFRα^fl/fl^ mice resulted in a significant reduction in myofibroblast scar thickness in the fibrotic rim 7 dpi compared with littermate controls in which PDGFRα had not been ablated (GFAP-Cre^–^;PDGFRα^WT/WT^, GFAP-Cre^+^;PDGFRα^WT/WT^, GFAP-Cre^–^;PDGFRα^fl/fl^) (Figure 5, F–H). Interestingly, the astroglial scar was unaffected in the GFAP-Cre^+^;PDGFRα^fl/fl^ mice, suggesting that the astroglial scar and the myofibroblast scar arise from different progenitors. Thus, loss of PDGFRα signaling in perivascular cells was sufficient to diminish myofibroblast expansion.

### Specific inhibition of PDGF-CC/PDGFRα signaling reduces stroke lesion volume and myofibroblast expansion in the fibrotic scar.

**Figure 6:**
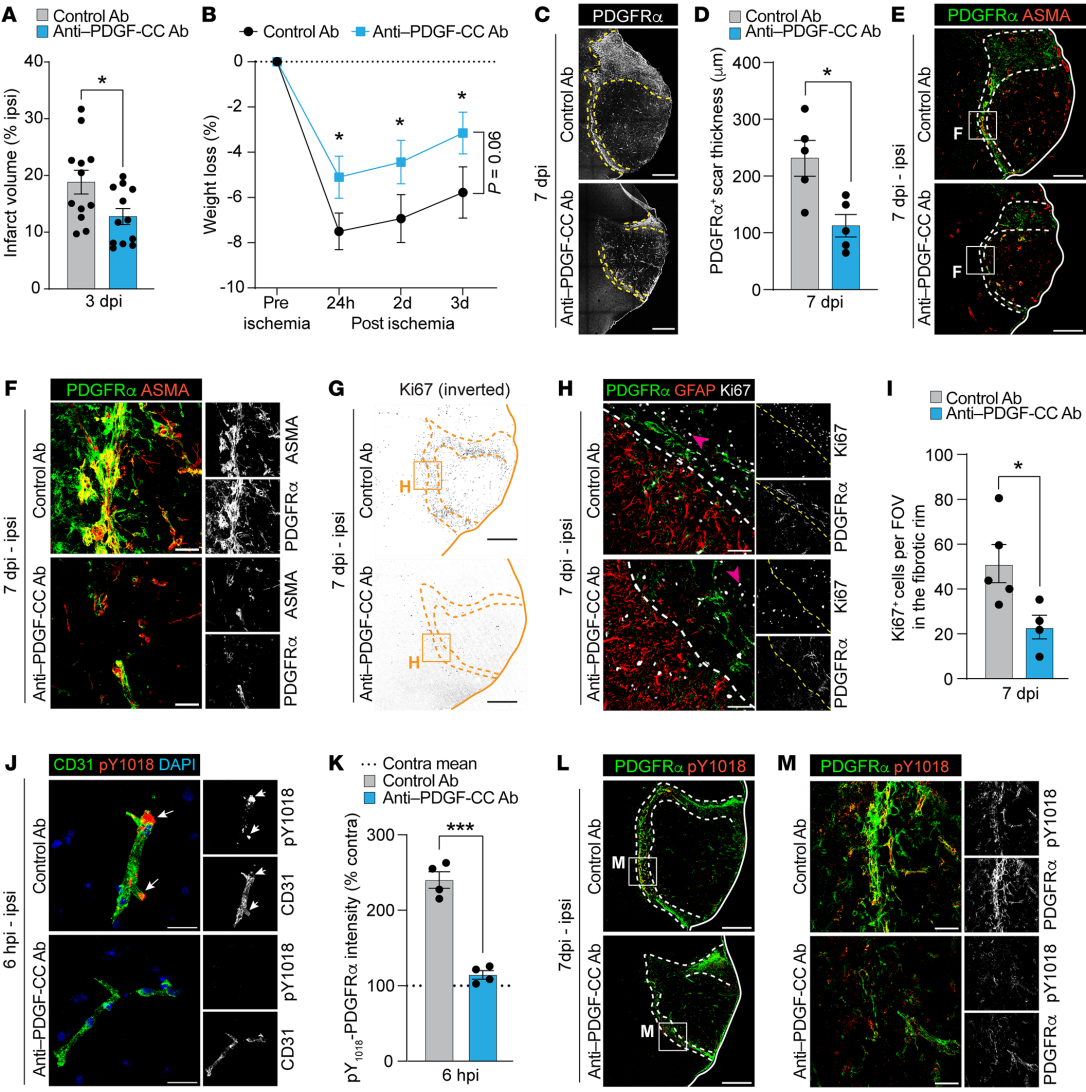
Anti–PDGF-CC antibody treatment reduces infarct volume and myofibroblast expansion in the fibrotic rim after MCAO. (**A**) Infarct volume 3 dpi (*n* = 12). (**B**) Weight during the first 3 dpi (*n* = 12). (**C**–**M**) Representative images of immunofluorescence staining and quantification in brain sections from control and anti–PDGF-CC antibody–pretreated mice collected at 6 hpi to 7 dpi. (**C**) Ipsilateral overviews of PDGFRα staining at 7dpi. (**D**) Quantification of PDGFRα^+^ scar thickness in the fibrotic rim (demarcated in **C**) (*n* = 5). (**E**–**H**) Costaining of PDGFRα and ASMA (**E** and **F**) and PDGFRα, GFAP, and Ki-67 (**G** and **H**) at 7 dpi. Arrowheads: proliferating PDGFRα^˗^ cells. (**I**) Quantification of Ki-67^+^ nuclei in the fibrotic rim (*n* = 4–5). FOV, field of view. (**J**) High-magnification images from the ischemic area at 6 hpi of staining for phospho-PDGFRα (pY1018) and CD31. Arrows: phosphorylation of perivascular PDGFRα. (**K**) Quantification of perivascular phospho-PDGFRα expression in the ischemic area at 6 hpi (*n* = 4). (**L** and **M**) Costaining for phospho-PDGFRα (pY1018) and total PDGFRα at 7dpi. Stitched epifluorescent tiles (**C**) and single-plane (**E**, **G**, and **L**)/maximum-intensity projections (**F**, **H**, **J**, and **M**) of confocal images. Dashed lines demarcate the myofibroblast scar (**C**, **E**, **G**, and **L**) and the glial border (**H**). Data points represent individual animals; bars, group mean ± SEM (**A**, **D**, **I**, and **K**); in **B** data points represent group mean ± SEM. Dashed line in **K** shows contralateral group mean. Two-tailed, unpaired *t* test with Welch’s correction (**A**, **D**, **I**, and **K**); 2-way repeated-measures ANOVA with Tukey’s post-hoc test (**B**). *P < 0.05; ***P < 0.001. Scale bars: 500 μm (**C**, **E**, **G**, and **L**); 50 μm (**F**, **H**, and **M**); 20 μm (**J**).

To further investigate the role of PDGFRα in myofibroblast expansion after MCAO, we made use of a monoclonal anti–human PDGF-CC antibody that neutralizes PDGF-CC, the ligand for PDGFRα, in genetically modified mice expressing a humanized form of PDGF-CC (PDGF-CC^hum^) (25, 26). This allowed us to exclusively inhibit PDGF-CC/PDGFRα signaling, without affecting PDGFRβ signaling or any of the other targets of imatinib. We found that at 3 dpi, infarct volume was significantly reduced in the animals receiving anti–PDGF-CC antibody treatment compared with animals treated with the control antibody (Figure 6A). This coincided with reduced weight loss, indicative of a better general condition, in anti–PDGF-CC antibody–treated animals (Figure 6B). Assessment of myofibroblast scar thickness at 7 dpi showed a significant reduction in animals treated with the anti–PDGF-CC antibody compared with controls (Figure 6, C–F). As with imatinib, the astroglial scar (Supplemental Figure 7, A and B) and the NG2 glial scar (Supplemental Figure 7, C and D) appeared to be unaffected by anti–PDGF-CC antibody treatment. Staining for Ki-67, we found extensive proliferation 7 dpi in the fibrotic rim of control antibody–treated mice, which was significantly reduced in anti–PDGF-CC antibody–treated animals (Figure 6, G–I, and Supplemental Figure 7E). To determine whether the effect of anti–PDGF-CC antibody treatment on the myofibroblast scar was mediated via PDGFRα, we assessed PDGFRα activation at 6 hpi and 7 dpi by immunofluorescence staining using 2 different antibodies that recognize specific phosphorylation sites on activated PDGFRα (pY754 and pY1018) (36, 41). Both antibodies detected phosphorylation of PDGFRα around vessels at 6 hpi in control animals, which was significantly reduced in animals treated with the anti–PDGF-CC antibody compared with the controls (pY1018, Figure 6, J and K, and Supplemental Figure 7F; pY754, Supplemental Figure 7, G–I). At 7 dpi, a strong PDGFRα phosphorylation signal was detected in the PDGFRα^+^ fibrotic rim of control-treated animals, whereas this was markedly reduced in anti–PDGF-CC–antibody treated animals (pY1018, Figure 6, L and M, and Supplemental Figure 7, J and K; pY754, Supplemental Figure 7, L and M). In summary, these data show that selective inhibition of the PDGF-CC/PDGFRα signaling pathway resulted in a reduction in stroke infarct volume and myofibroblast expansion similar to that seen with imatinib treatment, thus potentially offering a targeted treatment approach for patients with ischemic stroke.

### Imatinib progressively improves function in a sensory-motor integration test after MCAO.

**Figure 7:**
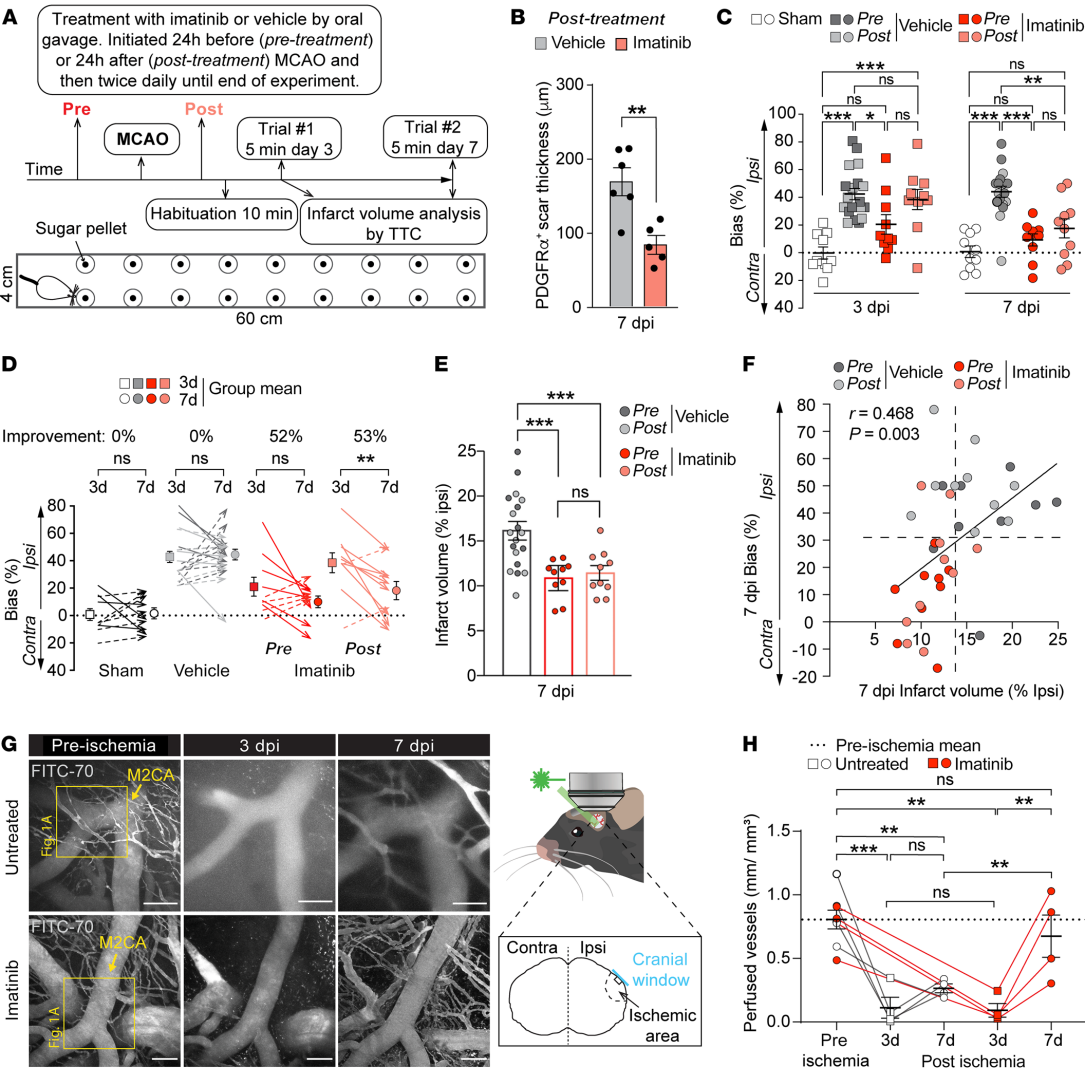
Imatinib progressively improves functional recovery after MCAO. (**A**) Schematic illustration of experimental design and the corridor task. The pellet explorations made from the left (ipsilateral to the lesion) or right (contralateral) side were counted. (**B**) Quantification of PDGFRα^+^ scar thickness in the fibrotic rim in imatinib-posttreated mice at 7 dpi (*n* = 5–6). (**C**) Exploration bias in mice pre- and posttreated with vehicle or imatinib, as well as in sham-operated mice (*n* = 10–19). (**D**) Change in exploration bias between 3 and 7 dpi. Arrows: individual mice (*n* =10–19). (**E**) Infarct volume at 7 dpi of vehicle-treated and imatinib-pretreated or posttreated mice (*n* = 10–19). (**F**) Correlation of infarct volume with exploration bias at 7 dpi (*n* = 9–10). (**G**) Representative maximum-intensity 2-photon images of FITC70 signal before (pre) and at different time points after (post) ischemia. (**H**) Quantification of vascular perfusion as assessed by intraluminal FITC70 signal using longitudinal 2-photon microscopy (*n* = 4). Data points represent individual animals; bars, group mean ± SEM (**B**, **C**, **E**, **F**, and **H**); in **D** arrows represent individual animals, and data points represent group mean ± SEM. Two-tailed, unpaired *t* test with Welch’s correction (**B**); 2-way repeated-measures ANOVA with Tukey’s post-hoc test (**C**, **D**, and **H**); 1-way ANOVA with Welch’s test (**E**); linear regression (**F**). *P < 0.05; **P < 0.01; ***P < 0.001. Scale bar: 100 μm (**G**).

To test how reduced BBB breach and myofibroblast expansion, after pre- or posttreatment with imatinib, affects functional recovery, we assessed a lateralized sensory-motor integration test at 3 and 7 dpi using the corridor task modified for mice (30, 42, 43) (see experimental outline in Figure 7A). This test is based on the fact that unilateral brain lesions will cause contralateral neglect and thus lead to a preference for exploring/retrieving objects or food placed on the side ipsilateral to the lesion (ipsilateral bias). Functional improvement after a treatment will therefore result in reduced ipsilateral bias. Analysis of the PDGFRα^+^ scar in *post-infarct-treated* mice, which received their first dose of imatinib 24 hours after MCAO and thus had experienced the first wave of MCAO-induced BBB leakage, revealed that *posttreatment* still resulted in a significant reduction in PDGFRα^+^ scar thickness (Figure 7B and Supplemental Figure 8A). The reduction in PDGFRα^+^ scar thickness was equal to the effect seen after imatinib *pretreatment* (Figure 4D), indicating that scar expansion was largely independent of the early BBB disruption. Using the corridor test, we found that at 3 and 7 dpi, all vehicle-treated mice preferentially explored sugar pellets on the side ipsilateral to the lesion (Figure 7C and Supplemental Video 1) and did not display any functional recovery over time (Figure 7D). Imatinib *posttreatment* significantly reduced ipsilateral exploration bias compared with vehicle control at 7 dpi, but not at 3 dpi (Figure 7C). Imatinib-*pretreated* animals on the other hand, displayed significantly reduced ipsilateral bias already at 3 dpi that was further improved at 7 dpi (Figure 7C). Further analysis showed that both imatinib-*pretreated* and -*posttreated* animals displayed about 50% functional improvement between 3 and 7 dpi (Figure 7D). Analysis of infarct volume at 7 dpi revealed that imatinib-*pretreated* and -*posttreated* animals displayed similar lesion sizes (Figure 7E) and that infarct size significantly correlated with exploration bias (Figure 7F). This correlation between infarct size and exploration bias is further supported by the fact that no difference was detected in either infarct volume (Supplemental Figure 8B) or exploration bias (Figure 7C) when imatinib-*posttreated* animals were compared with vehicle controls at 3 dpi. Using 2-photon microscopy, we followed vascular perfusion over time in the ischemic area of untreated and imatinib-*pretreated* animals from before onset of ischemia to 7 dpi (Figure 7, G and H). Our analyses showed that while there was no improvement in vessel perfusion between 3 and 7 dpi in control animals, vessel perfusion increased significantly in the imatinib-treated cohort in the same time frame (Figure 7H). This correlated well with exploration bias (Figure 7C), thus suggesting that vessel perfusion might contribute to the improvement in functional outcome seen in imatinib-treated animals. However, it should be noted that at 3 dpi, we detected no difference in vascular perfusion when comparing untreated and imatinib-*pretreated* animals (Figure 7H), even though functional improvement was detected following *pretreatment* with imatinib at this time point (Figure 7C). Taken together, these results suggest that late and continued intervention to block the PDGF-CC/PDGFRα signaling pathway might improve functional recovery by limiting myofibroblast scar formation.

## Discussion

We have previously shown that PDGF-CC/PDGFRα signaling regulates cerebrovascular permeability in a tPA-dependent manner and that inhibiting disease-induced BBB breakdown by targeting this pathway with imatinib significantly improves outcome in a number of experimental disease models (5–11). Yet how targeting the BBB translates into improved neurological outcome is poorly understood, and the current literature indicates a complex relationship between disease-induced BBB breakdown and CNS repair (4, 17, 44). Here we present data indicating that imatinib pretreatment (administered *before* induction of ischemia and disease-induced BBB breach) accelerated functional recovery after MCAO compared with imatinib posttreatment (administered 24 hours *after* induction of ischemia and the first peak of disease-induced BBB breach had subsided). Importantly, though, despite the fact that posttreatment was not targeting the first wave of BBB breach or the acute reactive gliosis response, imatinib-posttreated mice reached the same level of functional recovery as imatinib-pretreated mice at 7 dpi. Our data thus indicate a much-sought-after therapeutic strategy that could help restore brain function in the post-acute phase of ischemic stroke when current treatment options are limited to rehabilitation therapies.

We found that in addition to regulating BBB integrity, imatinib dampened MCAO-induced expression of profibrotic/proinflammatory genes in the cerebrovasculature and diminished myofibroblast expansion in the rim of the fibrotic scar after MCAO. This is of particular interest considering that the fibrotic component of the CNS scar has recently emerged as a potential target in CNS repair, and that a moderate and region-specific reduction in fibrotic scarring, which still allows wound closure, promotes axon regeneration and functional recovery in experimental models of spinal cord injury (SCI) (45) and multiple sclerosis (experimental autoimmune encephalomyelitis [EAE]) (46). Based on this, we hypothesize that modulation of the myofibroblast response, by targeting signaling via PDGFRα, contributes to the beneficial effect seen on neurological and functional outcome with imatinib after ischemic stroke (9, 15). This is supported by a line of evidence including studies showing that PDGF-CC/PDGFRα signaling is associated with fibrotic and myofibroblast expansion in other organs (23, 47–51) and that ablation of PDGFRα in myofibroblast progenitor cells reduces myofibroblast differentiation and improves liver function in a model of liver disease (49), while sustained myofibroblast expansion is detrimental to healing processes (21). Importantly, we found that TGF, which is a key factor stimulating myofibroblast differentiation (21), was upregulated in the cerebrovasculature following PDGF-CC administration and downregulated following imatinib treatment in disease-associated microglia/macrophages. Last, our data show that imatinib reduced MCAO-induced expression of the canonical myofibroblast gene fibronectin. Fibronectin is known to decrease neuronal growth cone velocity in vitro (52) and contribute to remyelination failure within multiple sclerosis lesions (53), thus suggesting that a fibronectin-dense scar might interfere with axon regeneration, while decreased fibronectin deposition could ameliorate functional recovery. Further experiments will, however, be needed to fully understand the functional contribution of the myofibroblast scar to CNS recovery and to delineate the exact mechanism by which PDGF-CC/PDGFRα signaling is involved in this process.

The proposed progenitors of myofibroblasts include circulating bone marrow–derived fibrocytes (54, 55); tissue-resident/meningeal/choroid-plexus fibroblasts (56), and other mesenchymal cells related to blood vessels, e.g., pericytes, that could differentiate via EMT/EndoMT (40). In the CNS, lineage tracing analyses largely supports the latter, i.e. that perivascular cells in the NVU are the main progenitors of the fibrotic (39, 57) and myofibroblast scar formation (58), of which a subset resides along medium- to large-sized vessels and expresses PDGFRα and PDGFRβ. These studies suggest that activated perivascular cells leave the vessel wall in the acute phase following ischemia, after which they migrate/proliferate/differentiate to form the myofibroblast scar in the chronic phase after ischemia. Thus, blocking the initial activation of the perivascular cells would stop them from leaving the vessel wall, leading to fewer cells that could proliferate/differentiate and populate the myofibroblast scar. Our data suggest the existence of subpopulations of myofibroblasts within the fibrotic rim, of which some myofibroblasts expressed high levels of PDGFRα (PDGFRα^hi^PDGFRβ^hi^), while others displayed low expression (PDGFRα^lo^PDGFRβ^hi^). Myofibroblasts with high expression levels of PDGFRα localized adjacent to the astroglial scar, while those with low expression levels of PDGFRα were found closer to the fibrotic core. It is possible that these subpopulations arise from multiple progenitors and that they exert different effects in the healing response after ischemic stroke. This is supported by recent findings showing that myofibroblasts arise from multiple progenitors after SCI (58) and that different subpopulations of myofibroblasts elicit different protective/harmful functions in repair processes (59). It is also possible, however, that the PDGFRα^lo^PDGFRβ^hi^ and PDGFRα^hi^PDGFRβ^hi^ cells represent different stages of myofibroblast maturation. Thus, imatinib might block differentiation into fully maturated myofibroblasts, while the number of progenitor cells remains the same. Understanding the temporospatial regulation as well as the cellular origin/potential multilineage differentiation of the injury-induced myofibroblasts is central to allowing for future manipulation of the fibrotic scar.

The cellular identity of the perivascular PDGFRα^+^ cells has not been completely elucidated, and these cells have been referred to in the literature as fibroblast-like cells (56, 60), type A pericytes (57), and perivascular stromal cells (61) and by us as perivascular astrocytes (based on coexpression with GFAP and AQP4) (7–10, 30, 36). Whether these names are referring to the same population or different subpopulations of cells remains to be determined. Nevertheless, our studies, utilizing GFAP-Cre;PDGFRα floxed mice — which display a greater than 60% reduction in perivascular PDGFRα signal in the brain (7) — or a monoclonal anti–PDGF-CC antibody (25) clearly demonstrate that genetic ablation of PDGFRα in GFAP-positive cells or pharmacologic neutralization of the ligand for PDGFRα results in a diminished myofibroblast scar. This suggests that PDGFRα signaling is critical for the expansion of the myofibroblast scar. In contrast, our analyses suggests that PDGFRβ is also inhibited by imatinib and constitutes a marker of myofibroblasts/their progenitors, it does not appear to drive the expansion of the myofibroblast scar. However, since PDGFRβ is coexpressed in the potential perivascular PDGFRα^+^ progenitor cells and myofibroblasts, and PDGF-CC can induce signaling via the heterodimeric receptor PDGFRαβ, it is tempting to speculate that signaling via PDGFRαα homodimers and PDGFRαβ heterodimers might be of importance for myofibroblast expansion, but that they might trigger distinct cellular responses.

In line with previous work (37), we detected a great number of TGF-β–expressing CD68^+^ cells in the ischemic area, presumably microglia/macrophages, although it should be noted that bone marrow–derived fibrocytes also express CD68 (54) and are capable of acquiring myofibroblast characteristics (55). Our data demonstrate that imatinib reduced expression of TGF-β in these CD68^+^ cells and attenuated MCAO-induced cerebrovascular expression of *Itgax* (encoding CD11c). Since TGF-β is a key regulator of myofibroblast differentiation (21) and *Itgax* is a well-known disease-associated microglia gene (62), our data imply direct immune system–fibrosis crosstalk in ischemic stroke. In support of this are studies demonstrating that macrophages promote myofibroblast expansion in skin repair (63) and that fibroblasts coordinate neuroinflammation after brain injury (64). Further, TGF-β–PDGFRα crosstalk (22) and macrophage-expressed PDGF-CC (65) have been implicated in myofibroblast scar expansion in other organs, and we found that efficient proteolytic activation of PDGF-CC requires the integrin Mac1 on microglia (36). The fibrotic scar has been shown to play a role in regulating disease severity during neuroinflammation in EAE (46), and interestingly, we have previously shown that imatinib and anti–PDGF-CC antibody treatment can ameliorate EAE severity (6, 11). Originally we ascribed the beneficial effect of anti–PDGF-CC/PDGFRα treatment in EAE to the attenuation of disease-provoked BBB breakdown, but it is possible that inhibiting the PDGF-CC/PDGFRα pathway also targets the fibrotic response in EAE.

Taken together, our results provide evidence of what we believe to be a novel way to modify the myofibroblast scar by targeting PDGFRα signaling, either with imatinib or with a monoclonal anti–PDGF-CC antibody. Importantly, our findings demonstrate improved functional recovery following subacute administration of imatinib, which might be of great importance given the current lack of post-acute treatment options for patients with ischemic stroke.

## Methods

### Sex as a biological variable.

Sex as a biological variable was considered by making use of both male and female animals. Findings were similar for both sexes.

### Animals.

WT C57BL/6 (Charles River), C57BL/6NTac-Pdgfc^tm3633(K242T, K246R, R299S, K318R, N342S, A343T)Arte^ (referred to as PDGF-CC^hum^ mice) (26), GFAP-Cre;PDGFRα^flox^ (7), and Cldn5(BAC)-GFP mice (24, 60, 66), aged 2–7 months, were used in this study. PDGF-CC^hum^ mice express a humanized growth factor domain that allows neutralizing PDGF-CC signaling using a murine anti–human PDGF-CC antibody (25, 26). GFAP-Cre;PDGFRα^flox^ mice express Cre-recombinase under the murine GFAP promoter (67) and loxP sites flanking exons 2 and 3 of the PDGFRα gene (68). Cldn5(BAC)-GFP mice express cytosolic GFP under the CLDN5 promoter, leading to endogenous labeling of endothelial cells.

### Experimental ischemic stroke model.

A photothrombotic model of MCAO, using the photoactivatable dye rose bengal and light activation with a laser at the level of the surgically exposed middle cerebral artery (MCA), was used for experimental ischemic stroke. Details are presented in Supplemental Methods.

### Imatinib treatment.

Mice were treated with imatinib (Gleevec, Novartis; or generic version Mylan) by oral gavage (details on the preparation are presented in Supplemental Methods). Mice *pretreated* with imatinib received 3 doses (morning-night-morning) before MCAO and were then treated twice daily until the end of the experiment. In *posttreated* animals, imatinib treatment was initiated 24 hpi and then treated twice daily until the end of the experiment. As controls, mice were gavaged at the corresponding times with vehicle (H_2_O for the functional corridor tests; PBS for all other experiments), except for the 2-photon imaging experiments, in which the control animals were untreated.

### Anti–PDGF-CC antibody treatment.

Homozygous PDGF-CC^hum^ mice were intraperitoneally injected with a single dose of 2 mg/mL of either murine anti–human PDGF-CC (mu6B3, a gift from Paracrine Therapeutics described in ref. 25) or control antibody (IgG2a clone C1.18.4, BioXcell) in PBS (resulting in 400 µg per mouse) the day before MCAO induction. For experiments longer than 3 days, a second dose was given 3 dpi.

### Two-photon imaging.

Cranial window implantation over the cortical branch of the middle cerebral artery, stroke induction through the cranial window, 2-photon imaging, and image analysis were performed as previously described (24) (details are presented in Supplemental Methods).

### EB dye extravasation.

BBB breakdown in the ipsilateral hemisphere at different time points after MCAO was assessed by extravasation of EB dye as previously described (9, 36, 69) (details are presented in Supplemental Methods).

### Immunohistochemistry.

Tissue preparation of fixed or fresh-frozen brains for sectioning, immunofluorescence staining, and confocal image acquisition were conducted using standard protocols. Details, lists of antibodies, and image analyses are presented in Supplemental Methods.

### ICV injection.

Vehicle or active PDGF-CC was injected into the left lateral ventricle of naive C57BL/6 mice. Four hours after ICV, brains were rapidly dissected out and used for isolation of cerebrovascular fragments. Details are presented in Supplemental Methods.

### Isolation of cerebrovascular fragments, generation of mRNA, and real-time qPCR analysis.

Cerebrovascular fragments were isolated with antibody-coupled magnetic beads as previously described (30, 70). RNA was extracted and used for mRNA expression array analysis and cDNA generation for real-time qPCR analysis. Methodological details and a list of primers are presented in Supplemental Methods.

### Microarray and data analysis.

GeneChip ST Arrays (GeneChip Mouse Gene 2.0 ST Array) were hybridized with cDNA from cerebrovascular fragments and washed, stained, and scanned. Differential gene expression for molecules from the dataset that met the log_2_ fold change of greater or less than 0.05 and *P* value less than 0.05 cutoff in cerebrovascular fragments from imatinib- and vehicle-treated mice were compared using the Ingenuity Pathway Analysis platform (QIAGEN). The molecules in this dataset were grouped by biological functions and/or diseases or were associated with a canonical pathway in Ingenuity’s knowledge base. To compare our dataset with the Harmonizome database (31), we used the dataset “fibrosis, CTD Gene-Disease Associations” and compared our dataset with all genes from the fibrosis dataset showing a standardized value higher than 1.5. Details are presented in Supplemental Methods.

### Corridor task and stroke volume.

Assessment of functional recovery by measuring lateralized sensory-motor integration using a corridor task was done as previously described (30). Infarct volume analysis with 4% 2,3,5-triphenyltetrazolium chloride (TTC) staining was performed as described previously (9, 36).

### Statistics.

Data analysis was performed using GraphPad Prism 9 statistical software. For statistical analysis in experiments with only 2 groups, a 2-tailed, paired or unpaired *t* test with Welch’s correction was used. For experiments with more than 2 groups, statistical evaluation correcting for multiple comparisons and/or repeated measures was performed as stated in the figure legends, with statistical significance defined as *P* < 0.05. Data are presented as mean ± SEM.

### Study approval.

All experiments in this study were approved and performed in accordance with the guidelines of the Swedish National Board for Laboratory Animals and the European Community Council Directive (86/609/EEC) and were approved by the North Stockholm Animal Ethics Committee and the Institutional Animal Care and Use Committee Unit for Laboratory Animal Medicine at the University of Michigan.

### Data availability.

Data are available in the Supporting Data Values file. Raw data of the microarrays have been deposited in the NCBI’s Gene Expression Omnibus database (GEO GSE137534).

## Author contributions

LF and JP designed the study and wrote the manuscript with critical input from MZ and DAL and all the coauthors; UE contributed critical resources; JP, MZ, CS, DT, MZA, KM, SJIR, SAL, LM, IN, EJS, and LF conducted experiments and acquired data; JP, MZ, CS, DT, MZA, IN, and LF analyzed data.

## Supplementary Material

Supplemental data

Supplemental table 1

Supplemental table 2

Supplemental video 1

Supplemental video 3

Supplemental video 4

Supporting data values

## Version 1 01/14/2025

In-Press Preview

## Version 2 03/03/2025

Electronic publication
