# Supplementary material for: PDGFRα inhibition reduces myofibroblast expansion in the fibrotic rim and enhances recovery after ischemic stroke
Source: J Clin Invest. 2025 Jan 14;135(5):e171077. doi: 10.1172/JCI171077 (PMC11870733; doi:10.1172/JCI171077)
Supplement: Supplemental data [file jci-135-171077-s257.pdf]

Supplementary Materials and Methods

Animal housing

Animals were housed in the animal facility at Karolinska Institutet (Stockholm, Sweden) in a climate-controlled environment in individually ventilated polystyrene cages containing aspen wood shavings and cage enrichments. Animals had free access to standard rodent chow and water and were subjected to a regulated 12 h light/dark cycle. Mice were backcrossed to the C57BL/6 background (Charles River) for a minimum of 3 generations.

Experimental ischemic stroke model

Prior to the experiment, mice were randomized within cages, by weight and treatment. For ischemic stroke induction mice were anesthetized with isoflurane and securely placed under a dissecting microscope. For pain treatment 5 mg/kg carprofen (Rimadyl Vet.®, Pfizer) and 0.06 mg/kg buprenorphine (Temgesic, Indivior UK Limited) were given subcutaneously (s.c.). The local analgesic lidocaine (Lidocaine Accord, Accord) was s.c. injected at the site of surgery. The left middle cerebral artery (MCA) was exposed and a laser Doppler flow probe (Type N (18 gauge), Transonic Systems) was placed on the surface of the cerebral cortex (1, 2). The probe was connected to a flowmeter (Transonic model BLF22) and relative tissue perfusion units (TPU) were traced. The photoactivatable dye Rose Bengal (Fisher Scientific) was diluted to 10 mg/ml in phosphate buffered saline (PBS) and then injected intravenously (i.v.) with the final dose of 40 mg/kg. A 3.5 mW green light laser (540 nm, Melles Griot) was directed at the MCA from a distance of 6 cm immediately after the injection of Rose Bengal. Total MCA occlusion (MCAO) was achieved when the TPU had dropped to less than 30% of pre-occlusion levels and to achieve a stable clot the laser was left on for 20 minutes. As controls, mice from the same cohort were shaved, skin was cut, the muscle retracted and either laser only or Rose Bengal only was used (sham operated mice). To alleviate postoperative pain, 5 mg/kg carprofen was administered by s.c. injection 24 h after the procedure (or up to 3 days if needed).

Imatinib treatment

Imatinib tablets were crushed into a fine powder, solubilized in vehicle, vortexed and incubated at 37°C in a water bath for 5 min. Insoluble components were spun down in a table microcentrifuge at 16,100 x *g* for 10 min. The supernatant was used for oral gavage performed with a steel gavage needle for mice to reach a daily dose of 250 mg/kg.

Cranial window implantation

The animals were anaesthetized with isoflurane, placed on a heating pad and head fixed in a surgical frame. The mice received 5 mg/kg carprofen, 0.06 mg/kg buprenorphine and 2 mg/kg dexamethasone (Dexadreson vet., MSD animal health) s.c.. Lidocaine was injected s.c. at the site of surgery before removing a flap of skin over the left hemisphere of the scull. A metal bar (split headpost small, Luigs & Neumann) was glued to the skull of the left hemisphere with super glue (Loctite, Henkel). The left temporal muscle was detached from the skull and a craniotomy of 3 mm in diameter was performed above the cortical branch of the MCA (M2CA). A glass coverslip (3 mm in diameter, #0, Multi Channel Systems GmbH) was placed into the opening and sealed with dental cement powder (Paladur) mixed in super glue. The surrounding tissue and metal bar were covered with dental cement. The animals received 5 m/kg carprofen and 2 mg/kg dexamethasone s.c. for the next 24 h (or up to 3 days if needed). The mice recovered for a minimum of ten days before starting the imaging protocol to allow the glial reaction to cease (3).

Photothrombotic stroke induction through the cranial window

The mice were anesthetized with isoflurane, placed on a heating pad and head-fixed in a surgical frame. 40 mg/kg Rose Bengal diluted in PBS were injected i.v.. A 3.5 mW cold green light laser was pointed on the M2CA for 20 min through the cranial window. Using a Doppler flow probe connected to a flowmeter, relative TPU was measured. When the TPU was less than 30% of its original value, occlusion of the M2CA (M2CAO) was assumed.

Two-photon imaging and image analysis

The anesthetized animals were imaged before stroke induction (pre ischemia), immediately after (1-2 hours post ischemia (hpi), 24 hpi, 2 days post ischemia (dpi), 3 dpi as well as 7 dpi). Blood flow was visualized by i.v. injection of 100 µl of 10 mg/ml fluorescent dextran (FITC70, TdB Labs) prior to imaging. The two-photon microscope (TSC SP8 multiphoton system, Leica) was equipped with a 25x/1.0 NA water immersion objective (motCORR VISIR, Leica) heated to 37°C by an objective mantle heater (ALAOBJ heater and Scientifica). A pulsed Ti:Sapphire infrared laser (Chameleon S, Coherent) was used at the wavelengths of 920 nm to excite FITC70 and 880 nm to excite Cldn5-GFP. The signal was filtered with a 525/50 nm band-pass filter (Semrock) and collected with a photo-multiplier tube (Leica). For image acquisition, the LAS X software (Leica) was used. Z-stacks of 591 µm x 591 µm x 1 µm (xyz) covering 100-300 µm depth, at 1024 x 1024 pixel resolution were acquired. Image analysis was conducted as previously described (30), details can be found in Supplementary Table 1.

Supplementary Table 1: Analysis of two-photon images

Figure	Experiment	Images	Number of images per animal	z-depth (µm)	Analysis method
**1 B**	FITC70 extravasation	two-photon z-stacks	2 per time point and animal	100-200	Intra-/extraluminal segmentation of FITC70 signal with Simple Neurite Tracer (4) in Fiji/ ImageJ as described in (5)
**1 D, S1 J-L**	Vessel diameter	two-photon z-stacks	3 per time point and animal	100-300	Manual measuring of vessel diameter pre and post M2CAO in Fiji/ImageJ
**7 H**	Vessel perfusion	two-photon z-stacks	2 per time point and animal	100-200	Semi-automated vessel tracing with Simple Neurite Tracer (4) in Fiji/ ImageJ as described in (5)

Evans blue (EB) dye extravasation

For analysis of cerebrovascular permeability after MCAO, stroked mice were injected i.v. with 100 µl of 4% Evans blue (EB) dye (Sigma-Aldrich) 1 h prior to sacrifice. The animals were

transcardially perfused with PBS for 5 min under isoflurane anesthesia and the brains removed and photographed (Canon PowerShot SX200IS camera). Thereafter the brains were separated into hemispheres and each hemisphere was homogenized in N,N-dimethylformamide (Sigma-Aldrich) in Precellys lysing tubes and centrifuged twice at 16,100 x *g* for 20 min. The supernatants were collected, EB extravasation was determined by absorbance measurement and quantified separately in the ipsi- and contralateral hemispheres. Background EB level in the non-ischemic contralateral hemisphere was subtracted from the ischemic hemisphere. EB levels in each hemisphere were calculated using the following formula:

(A620nm – ((A500nm + A740nm) / 2)) / mg wet weight

Immunofluorescent stainings and confocal microscopy

Mice were anesthetized with isoflurane and transcardially perfused with PBS followed by fixation with 4% paraformaldehyde (PFA) in PBS. The brains were dissected and postfixed in 4 % PFA for 1 h at room temperature (RT) and then either cut in 50 µm vibratome sections or kept in 30% sucrose on 4°C overnight for cryopreservation in OCT. For fresh-frozen sections, mice were transcardially perfused with PBS and the dissected brains were instantly cryopreserved in OCT. 12-14 µm coronal brain sections were sectioned in a cryostat.

Fresh-frozen sections were post-fixed with 4% PFA in PBS on ice for 15 min. For stainings that required antigen retrieval, heat-induced antigen retrieval (HIER) in DAKO buffer was used for 15-30 min (see Supplementary Table 2). All cryosections were permeabilized and then blocked with DAKO blocking solution, followed by incubation over night at 4°C with primary antibodies diluted in blocking solution. Vibratome sections were permeabilized and blocked with 1% bovine serum albumin in 0.5% TritonX-100/PBS and stained free floating in 24-well plates. The primary and secondary antibodies used can be found in Supplementary Table 2 and 3, respectively. DAPI (4',6-Diamidino-2-Phenylindole, Dihydrochloride) was included in the last PBS wash to visualize the nuclei. Following immunofluorescent staining the sections were mounted using ProLong Gold Antifade reagent (Molecular Probes).

Supplementary Table 2: List of Primary Antibodies

Primary Antibody	Species	Dilution	HIER	Provider	Reference Number
Alpha Smooth Muscle Actin (ASMA)-Cy3	mouse	1:200	+/-	Sigma-Aldrich	C6198
(mouse) B220/CD45R	rat	1:200	-	R&D systems	MAB1217
(mouse) CD3	rabbit	1:200	-	Sigma-Aldrich	C7930
(mouse) CD11b	rat	1:50	-	BD Pharmingen	550282
(mouse) CD31	goat	1:200	+/-	R&D Systems	AF3628
(mouse) CD31	rat	1:50	+/-	BD Pharmingen	553370
CD68	rat	1:200	-	Biorad	MCA1957
Claudin5 (CLDN5)	rabbit	1:200	+	Zymed	34-1600
Collagen 1 (COL1)	rabbit	1:100	-	Abcam	ab21286
Fibronectin	rabbit	1:200	-	Sigma-Aldrich	F3648
Glial Fibrillary Acidic Protein (GFAP)	rat	1:200	+/-	Invitrogen	13-0300
(mouse) GFAP	rabbit	1:200	+/-	DAKO	Z0334
IBA1	rabbit	1:100	-	WAKO chemicals	019-19741
Ki67-EF660	rat	1:200	+/-	Thermo fisher	50-5698-98
Myelin Basic Protein (MBP)	rat	1:50	+	Merck	MAB386
(mouse) Myeloperoxidase (MPO)	rabbit	1:200	-	Abcam	ab208670
(rat) Neural/Glial antigen 2 (NG2)	mouse	1:100	+/-	Sigma-Aldrich	N8912
(rat) NG2	rabbit	1:200	+/-	Merck/Millipore	AB5320
Olig2	rabbit	1:100	+	Chemicon	AB9610
(mouse) Platelet-Derived Growth Factor Receptor alpha (PDGFRα)	goat	1:200	+/-	R&D Systems	AF1062
PDGFRβ	rabbit	1:50	+	Cell Signaling	3169S
phospho-PDGFRα (pY754)	rabbit	1:100	-	Cell Signaling	2992S
phospho-PDGFRα (pY1018)	rabbit	1:100	-	Cell Signaling	4547S
SOX9	rabbit	1:500	+	Merck/Millipore	AB5535
(mouse) TER119	rat	1:500	+	R&D systems	MAB1125
TGF beta 1 (3C11) conjugated to Alexa Fluor® 488	mouse	1:200	-	Santa Cruz	sc-130348 AF488

Table 3: List of Secondary Antibodies

Secondary Antibody	Species	Dilution	HIER	Provider
mouse (coupled to Alexa Fluor dyes)	donkey	1:500	+/-	Invitrogen

goat (coupled to Alexa Fluor dyes)	donkey	1:500	+/-	Invitrogen
rabbit (coupled to Alexa Fluor dyes)	donkey	1:500	+/-	Invitrogen
rat (coupled to Alexa Fluor dyes)	donkey	1:500	+/-	Invitrogen

Images were acquired at RT with a Zeiss LSM700 confocal microscope, a Leica SP8 inverted confocal microscope or a Zeiss Axio Observer Z1 inverted microscope and the ZEN 2009 software (Carl Zeiss Microimaging GmbH) or LASX (Leica) software for Zeiss or Leica microscopes, respectively. For all quantifications (see Supplementary Table 4 for details), images were acquired using the same settings (within the respective staining experiment) and taken in comparable anatomic positions for each animal. If maximum intensity projections were used for image analysis, the area covered by the preceding z-stack was of equal size in the control and treated animal group. The result from all fields of view (FOV) in a given animal was averaged to obtain the value for that individual. All images shown are representative of the respective staining and were processed and analyzed using Volocity 3D image analysis software (PerkinElmer), Adobe Photoshop CC or Fiji/ImageJ (National Institutes of Health).

Table 4: Quantification of immunofluorescent stainings

**Figure**	**Experiment**	**Images**	**Number of images per animal**	**z-depth (μm)**	**Analysis method**
1 G	PDGFRα$^+$ vessels (Imat pretreat)	confocal z-stacks	8	8	Manual counting in maximum intensity projection in Fiji/ImageJ
1 H	GFAP$^+$ vessels (Imat pretreat)	confocal z-stacks	8	8	Manual counting in maximum intensity projection in Fiji/ImageJ
3 C	GFAP intensity (Imat pretreat)	confocal single planes	6	-	Determination of area of antibody immunoreactivity above a set threshold in Fiji/ImageJ
3 F	NG2-glia cell body size (Imat pretreat)	confocal z-stacks	10	11	Measurement of cell body size of single PDGFRα$^+$ NG2-glia cells using Volocity
3 K	CD68$^+$ macrophages (Imat pretreat)	-	-	-	Manual cell counting while scanning in 4 FOVs

4 D	PDGFRα$^+$ scar thickness (Imat pretreat)	confocal z-stacks	Entire scar	10	Determination of scar thickness from 20 different positions along the scar (5 per image) in Fiji/ImageJ
4 I	Fibronectin intensity (Imat pretreat)	confocal single planes	5-6	-	Determination of area of antibody immunoreactivity above a set threshold in Fiji/ImageJ
4 L	ASMA$^+$ scar thickness (Imat pretreat)	Epifluorescent tiles	Entire scar	-	Determination of scar thickness from 20 different positions along the entire scar in Adobe Illustrator
5 E	PDGFRβ$^+$ scar thickness (Imat pretreat)	confocal z-stacks/ single planes	Entire scar	10 / -	Determination of scar thickness from 20 different positions along the entire scar in Adobe Illustrator
5 H	PDGFRα$^+$ scar thickness (GFAP^{cre};Ra^{Flox})	epifluorescent tiles	Entire scar	-	Determination of scar thickness from 20 different positions along the scar in Adobe Illustrator
6 D	PDGFRα$^+$ scar thickness (pre antiPC ab)	confocal single planes	Entire scar	-	Determination of scar thickness from 20 different positions along the scar in Adobe Illustrator
6 I	Ki67$^+$ cells (anti-PC Ab pretreat)	confocal single planes	4 per region	-	Manual counting of positive nuclei in Fiji/ImageJ
6 K	pY1018 intensity (anti-PC Ab pretreat)	confocal z-stacks	5 per region	5	Determination of area of antibody immunoreactivity above a set threshold (normalized to contralateral) in Fiji/ImageJ
7 B	PDGFRα$^+$ scar thickness (Imat posttreat)	epifluorescent tiles	Entire scar	-	Determination of scar thickness from 20 different positions along the scar in Adobe Illustrator
S1D	Microbleeds (Imat pretreat)	confocal single planes	16	-	Manual counting of microbleeds per FOV in Fiji/ImageJ
S1H	CLDN5 intensity (Imat pretreat)	confocal z-stacks	3-5	6	Determination of area of antibody immunoreactivity above a set threshold in Fiji/ImageJ
S1I	CD31$^+$ vessels >10 µm (Imat pretreat)	confocal z-stacks	5	6	Manual counting in maximum intensity projection in Fiji/ImageJ

S3C	SOX9$^+$ cells (Imat pretreat)	confocal z-stacks	20	9	Manual counting of positive nuclei in Fiji/ImageJ
S3G	MBP intensity (Imat pretreat)	confocal single planes	Entire ipsi-lateral	-	Determination of area of antibody immunoreactivity above a set threshold in Fiji/ImageJ
S3L	CD11b+ Microglia/ Macrophages (Imat pretreat)	-	-	-	Manual cell counting while scanning in 4 FOVs
S4B	Immune cell infiltration (Imat pretreat)	-	-	-	Manual cell counting while scanning in 4 FOVs
S5E	COL1 intensity (Imat pretreat)	confocal z-stacks	2	11	Determination of area of antibody immunoreactivity above a set threshold in Fiji/ImageJ
S5O	Ki67$^+$ intensity (Imat pretreat)	confocal single planes	6	-	Determination of area of antibody immunoreactivity above a set threshold in Fiji/ImageJ
S7I	pY754 intensity (pre antiPC ab)	confocal z-stacks	5	5	Determination of area of antibody immunoreactivity above a set threshold (normalized to contralateral) in Fiji/ImageJ

Intracerebroventricular injection (ICV)

3 µl of vehicle (PBS) or 3 µM active PDGF-CC were injected into the left lateral ventricle (stereotactic coordinates: bregma -0.6, mediolateral -1.2, and dorsoventral 2) of naïve C57BL/6 mice using a stereotax. 4 h after ICV, the mice were anesthetized with isoflurane and transcardially perfused with Hanks' balanced salt solution (HBSS). Brains were rapidly dissected out, placed into ice-cold HBSS and used for isolation of vascular fragments.

Isolation of cerebrovascular fragments and generation of mRNA

Cerebrovascular fragments were isolated from mice 3 hpi, 24 hpi or 7 dpi or from sham operated mice. Mice were anesthetized with isoflurane and perfused with HBSS. The brains were rapidly dissected, placed in ice-cold HBSS and mechanically and enzymatically dissociated. Biotin rat anti-mouse CD31 antibodies (BD Biosciences) coupled to magnetic Dynabeads (Dynabeads

biotin binder, Invitrogen) were used to pull out vascular fragments. Total RNA was extracted from both the wash and the eluate fractions using the RNeasy kit (Qiagen) and the QIAcube (Qiagen) including on column DNA-digestion for fully automated sample preparation. RNA concentration and purity were determined through measurement of A260/A280 ratios with a NanoDrop ND-1000 Spectrophotometer (NanoDrop Technologies). Confirmation of RNA quality was assessed using the Agilent 2100 Bioanalyzer (Agilent Technologies). mRNA was used for expression array analysis or cDNA generation for qPCR analysis. cDNA was prepared using the iScript kit (Bio-Rad). The purity of the cerebrovascular fragments and the fraction of various cell types in the preparations was analyzed by real time quantitative PCR (qPCR) using primers for endothelial cells (*Cldn5*, *Pecam1*), vascular mural cells (*Pdgfrb*), astrocyte endfeet (*Aqp4*), and neurons (*Dlg4*). The analysis revealed high enrichment of endothelial cells (187 ± 29 fold for *Cldn5* and 78 ± 16 fold for *Pecam1*), followed by vascular mural cells (25 ± 6 fold) and astrocyte endfeet (13 ± 2 fold). The cerebrovascular fragments were devoid of neurons (0.1 ± 0.05 fold). The values represent the enrichment factor (± S.E.M.) in the vascular fragment eluates compared to the wash fraction (rest of the brain tissue). These results indicate high purity of the cerebrovascular fragments.

Real-time PCR analysis

Real-time quantitative PCR was performed using KAPA SYBR FAST qPCR Kit Master Mix (2x) Universal (KAPA Biosystems) in Rotor-Gene Q (Qiagen) Real-time PCR thermal cycler according to the manufacturer's instructions. A list of the primers used can be found in Supplementary Table 5. Expression levels were normalized to the expression of *Rpl19*.

Table 5: List of primers

Gene	Forward (5'-sequence)	Reverse (5'-sequence)
Angpt2	AGAATACAAAGAGGGCTTCGGG	GACAAACTCATTGCCCAGCC
Aqp4	ATGGTGGATCCCACACCGAG	AGGCGGTGGGGTAAGTGTG
Ccl5	CCTGCTGCTTTGCCTACCTCTC	ACACACTTGGCGGTTCCTTCGA
Cldn5	GTGGAACGCTCAGATTTCAT	TGGACATTAAGGCAGCATCT
Cldn6	GGAACTCCAAGTCTCGTCTGGT	AGTCCTGGATGATAGAGTGGGC
Col5a2	GTGGCATAGGAGAGAAAGGTGC	GCCAACTAAGCCTCTAGGACCA
Cxcl10	ATCATCCCTGCGAGCCTATCCT	GACCTTTTTTGGCTAAACGCTTTC
Dlg4	CGCCCCCTCTGGAACACAGC	TGCTGGAGGGCGAAGAAAACCG
Fn1	CCCTATCTCTGATACCGTTGTCC	TGCCGCAACTACTGTGATTCGG
Fut11	CAGTCCCATTGCGATGTGCCTT	CTGTGTCCTGTAATCGCACGGT

Hpse	CTGTCCAACACCTTTGCAGCTG	CCACTAAGTGGTAGTTGCCTGC
Icam1	TGAGGTCCTTGCCTACTTGC	GCTTTGAGAACTGTGGCACC
Il1a	ATCAGCAACGTCAAGCAACG	AAGGTGCTGATCTGGGTTGG
Itgax	TGCCAGGATGACCTTAGTGTCG	CAGAGTGACTGTGGTTCCGTAG
Mmp9	TCATAGGCCCAGAGGTAACCC	AGTCGAATCTCCAGACACGC
Pecam1	TACTGGGCTTCGAGAGCATT	AGAGACGGTCTTGTCGCAGT
Pdgfc	GTGGAGGAAATTGTGCCTGT	TCCAGAGCCACATCAGTGAG
Pdgfra	TGGCATGATGGTCGATTCTA	CGCTGAGGTGGTAGAAGGA
Pdgfrb	GGAGTCCATAGGGAGGAAGC	CACCTTCTCCAGTGTGCTGA
Plat	GTTACACAGCGTGGAGGACCAA	CACGTCAGCTTTCGGTCCTTCA
Rpl19	GGTGACCTGGATGAGAAGGA	TTCAAGCTTGTGGATGTGCTC
Smad3	ATGAACCACAGCATGGACGC	ACATCGGATTCGGGGAGAGG
Tgfb	ACAGCCCTCGCACCCA	GCCACCGATCCAGACAGAGT
Vegfa	TCCAGGAGTACCCCGATGAGA	CACATCTGCTATGCTGCAGGA

Microarray and data analysis

10 ng of total RNA from each sample were used to generate amplified and biotinylated sense transcript cDNA from the entire expressed genome according to the Nugen Technologies, Inc. protocols Ovation® Pico WTA System V2 (M01224v2) and Encore Biotine Module (M01111v5). GeneChip® ST Arrays (GeneChip® Mouse Gene 2.0 ST Array) were hybridized for 16 h in a 45°C incubator, rotated at 60 rpm. According to the GeneChip® Expression Wash, Stain and Scan Manual (PN 702731 Rev 3, Affymetrix Inc.) the arrays were then washed and stained using the Fluidics Station 450 and finally scanned using the GeneChip® Scanner 3000 7G. The raw data were normalized in the free software Expression Console provided by Affymetrix (http://www.affymetrix.com) using the robust multi-array average (RMA) method first suggested by Li and Wong. Subsequent analysis of the gene expression data was carried out in the freely available statistical computing language R (http://www.r-project.org) using packages available from the Bioconductor project (www.bioconductor.org). In order to search for the differentially expressed genes between the different groups an empirical Bayes moderated t-test was applied, using the 'limma' package. To address the problem with multiple testing, the *P*-values were adjusted using the method of Benjamini and Hochberg.

To compare gene expression in vascular fragments from imatinib-treated mice versus vehicle controls 3 hpi and 24 hpi, molecules from the dataset that met the < or > log2 (0.5) fold change and *P*-value < 0.05 cutoff were uploaded to the ingenuity pathways analysis platform (Ingenuity Systems, www.ingenuity.com). The molecules in this dataset were grouped in biological

functions and/or diseases or were associated with a canonical pathway in Ingenuity's knowledge base. Right-tailed Fisher's exact test was used to calculate a *P*-value determining the probability that each biological function and/or disease assigned to that data set is due to chance alone. The significance of the association between the data set and the canonical pathway was measured in two ways: 1) A ratio of the number of molecules from the data set that map to the pathway divided by the total number of molecules that map to the canonical pathway is displayed. 2) Fisher's exact test was used to calculate a *P*-value determining the probability that the association between the genes in the dataset and canonical pathway is by chance alone. Raw data are deposited on the NCBI Gene Expression Omnibus database (accession no. GSE137534). To compare our dataset to the harmonizome database (6), we used the harmonizome dataset named "fibrosis, CTD Gene-Disease Associations". In the harmonizome database every gene included in a certain functional annotation has a standardized value indicating the relative strength of the association. Standardized values range from 1 to 2,88. We compared our dataset with all genes from the fibrosis dataset showing a higher standardized value than 1,5. The association with inflammation and metabolism was done manually and according to the current scientific knowledge.

Corridor task and stroke volume

To assess functional recovery, we measured lateralized sensory-motor integration using a corridor task adapted from (7, 8). Briefly, a plexiglass box (L=60 cm x W=4 cm x H=15 cm) was used, where ten pairs of adjacent Eppendorf caps, each containing 4-5 sugar pellets (20 mg per pellet; TestDiet), had been placed at 5 cm intervals. A corridor with the same dimensions but without adjacent Eppendorf caps was used for habituation. 24 hpi, imatinib or vehicle-treated mice were habituated to the corridor by scattering sugar pellets along the corridor floor and allowing them to freely explore for 10 min. Sham operated mice were used as controls. Lateralized sensory-motor integration was tested at 3 dpi and 7 dpi. On the testing day, mice were placed in the habituation corridor for 5 min in the absence of sugar pellets, then mice were transferred to one end of the testing corridor containing sugar pellets and video recorded for 5 min. All video recordings were analyzed by an investigator blinded to the experiment. A second investigator analyzed randomly selected videos independently to confirm scoring by the first investigator (approx. 16% of all videos were analyzed twice). The number of ipsilateral and contralateral explorations relative to the stroked hemisphere were counted until the mouse

made a total of 40 explorations or the video ended. An exploration was defined as a nose-poke into an Eppendorf cap, whether the sugar pellet was poked or eaten, and a new exploration was only counted by exploring a new cap. Data is expressed as ipsilateral bias, calculated as:

Ipsilateral bias (%) = ((ipsilateral (left) explorations contralateral (right) explorations) / total explorations (left+right))*100

Following the last day of functional testing at day 7 infarct volume analysis was performed as described previously (2, 9). Lesion volume at 3 dpi was also determined in a separate cohort of mice receiving post treatment with imatinib (initiated 24 hpi). Briefly, brains were removed at 7 dpi and 2 mm thick coronal sections of the whole brain were stained with 4% 2,3,5-triphenyltetrazolium chloride (TTC) in PBS for 20 min at 37°C and then fixed in 4% paraformaldehyde solution for 10 min. TTC stained sections were captured with an Olympus digital C-3030 color camera attached to an SZ-60 Olympus microscope. The sections were analyzed with Fiji/ImageJ using the following formula:

$V_{\%stroke}$ = ∑(lesion area)/∑(total area of ipsilateral hemisphere)*100, where $V_{\%stroke}$ is stroke volume calculated as percent of the ipsilateral hemisphere.

Limitations of the study

In humans, stroke severity and clinical prognosis can vary substantially, depending on the cause and site of the occlusion as well as the age and co-morbidities of the patient. Preclinical models of stroke cannot reproduce human disease in its entirety, for example due to the use of inbred mouse strains which do not display the same genetic variability, environmental and lifestyle factors that human patients do. Nonetheless, a variety of animal models for ischemic stroke exists and MCAO models are the most widely used models in ischemic stroke research (10).

Supplementary References

1. Fredriksson L, et al. Identification of a neurovascular signaling pathway regulating seizures in mice. *Annals of Clinical and Translational Neurology*. 2015;2(7):722-38.
2. Su EJ, et al. Activation of PDGF-CC by tissue plasminogen activator impairs blood-brain barrier integrity during ischemic stroke. *Nature Medicine*. 2008;14(7):731-7.
3. Holtmaat A, et al. Imaging of experience-dependent structural plasticity in the mouse neocortex in vivo. *Behav Brain Res*. 2008;192(1):20-5.
4. Longair MH, et al. Simple Neurite Tracer: open source software for reconstruction, visualization and analysis of neuronal processes. *Bioinformatics (Oxford, England)*. 2011;27(17):2453-4.
5. Protzmann J, et al. Analysis of ischemic stroke-mediated effects on blood-brain barrier properties along the arteriovenous axis assessed by intravital two-photon imaging. *Fluids Barriers CNS*. 2024;21(1):35.
6. Rouillard AD, et al. The harmonizome: a collection of processed datasets gathered to serve and mine knowledge about genes and proteins. *Database (Oxford)*. 2016;2016.
7. Grealish S, et al. Characterisation of behavioural and neurodegenerative changes induced by intranigral 6-hydroxydopamine lesions in a mouse model of Parkinson's disease. *Eur J Neurosci*. 2010;31(12):2266-78.
8. Dowd E, et al. The Corridor Task: a simple test of lateralised response selection sensitive to unilateral dopamine deafferentation and graft-derived dopamine replacement in the striatum. *Brain Res Bull*. 2005;68(1-2):24-30.
9. Su EJ, et al. Microglial-mediated PDGF-CC activation increases cerebrovascular permeability during ischemic stroke. *Acta Neuropathologica*. 2017;134(4):585-604.
10. Sommer CJ. Ischemic stroke: experimental models and reality. *Acta Neuropathol*. 2017;133(2):245-61.
11. Vanlandewijck M, et al. A molecular atlas of cell types and zonation in the brain vasculature. *Nature*. 2018;554(7693):475-80.

Supplementary Fig. S1. Protzmann et al.

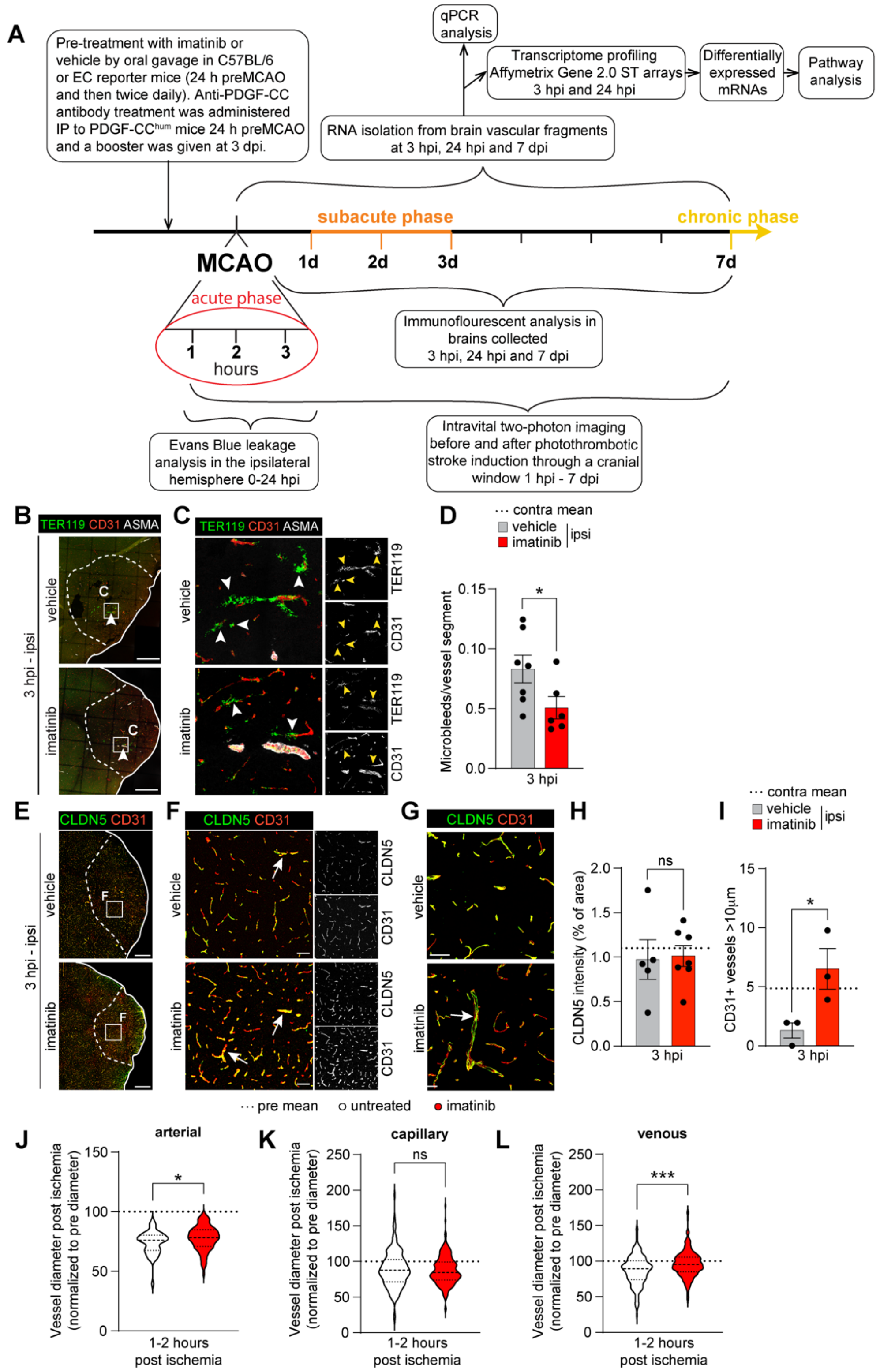

Supplementary Figure S1. Imatinib attenuates MCAO-induced microbleeds and vasoconstriction. (**A**) Schematic illustration of experimental design in Figure 1-6 and Supplementary Figure S1-S7. (**B-I**) Representative maximum intensity projections of confocal z-stacks of immunofluorescent stainings and quantification in brain sections from vehicle and imatinib-treated mice collected at 3 hours post ischemia (3 hpi). Ischemic area outlined with dashed lines. Ipsilateral overviews (**B**) and high-magnification images acquired in the ischemic area (**C**) from co-stainings for TER119, ASMA and CD31. Arrowheads, microbleeds. (**D**) Quantification of microbleeds (*N* = 6-7). Ipsilateral overviews (**E**) and high magnification images acquired in the ischemic area (**F** and **G**) from co-stainings for the tight junction protein claudin-5 (CLDN5) and the endothelial cell marker CD31. Arrows, > 10 µm diameter vessel. (**H**) Quantification of

CLDN5 expression based on antibody immunoreactivity intensity above a set threshold (*N* = 5-7). (**I**) Quantification of CD31 positive vessels with a diameter over 10 μm (arrows in **F** and **G**) (*N* = 3). Data points represent individual animals, bars the group mean ± SEM and the dashed line in **H** and **I** the contralateral group mean. (**J-L**) Longitudinal two-photon microscopy in endothelial reporter mice. Quantification of relative vessel diameter change in the arterial (**J**), capillary (**K**) and venous (**L**) segments 1-2 hpi compared to the diameter before onset. *N* = 46 (untreated) and 89 (imatinib) arteries/arterioles; 155 (untreated) and 249 (imatinib) capillaries; 198 (untreated) and 274 (imatinib) venules/veins from 4 (untreated) and 5 (imatinib) animals. Two-tailed, unpaired t-test with Welch's correction (**D** and **H-L**). Statistical significance: ns, non-significant; * $P < 0.05$; *** $P < 0.001$. Scale bars: 500 μm (**B** and **E**); 50 μm (**C**, **F** and **G**).

Supplementary Fig. S2. Protzmann et al.

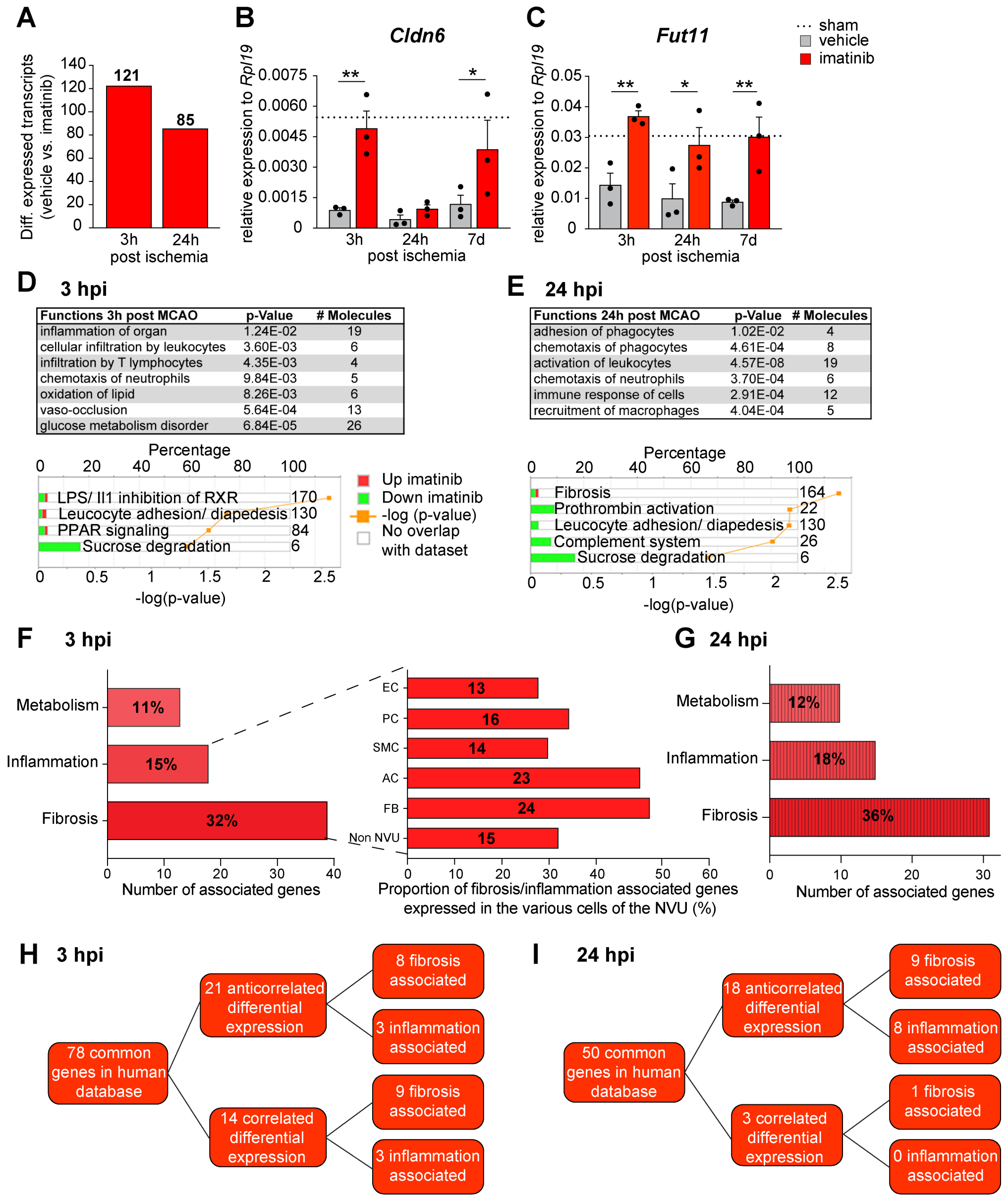

Functions 3h post MCAO	p-Value	# Molecules
inflammation of organ	1.24E-02	19
cellular infiltration by leukocytes	3.60E-03	6
infiltration by T lymphocytes	4.35E-03	4
chemotaxis of neutrophils	9.84E-03	5
oxidation of lipid	8.26E-03	6
vaso-occlusion	5.64E-04	13
glucose metabolism disorder	6.84E-05	26

Functions 24h post MCAO	p-Value	# Molecules
adhesion of phagocytes	1.02E-02	4
chemotaxis of phagocytes	4.61E-04	8
activation of leukocytes	4.57E-08	19
chemotaxis of neutrophils	3.70E-04	6
immune response of cells	2.91E-04	12
recruitment of macrophages	4.04E-04	5

Supplementary Figure S2. Imatinib affects expression of profibrotic/ proinflammatory transcripts in the cerebrovasculature after MCAO. (**A**) Differentially expressed genes in cerebrovascular fragments isolated from the ipsilateral hemisphere of imatinib-pretreated mice and vehicle controls 3 and 24 hours post ischemia (hpi). Cutoff: *P* < 0.05 and log2 (0.5) fold change (*N* = 2-3). (**B** and **C**) Confirmation by qPCR of two of the top regulated genes detected in our BBB transcriptome analysis in **A** (*N* = 3). (**D** and **E**) Ingenuity pathway analysis of the differentially expressed genes in the ischemic cerebrovasculature of imatinib pretreated mice compared to vehicle controls at 3 hpi (**D**) and 24 hpi (**E**). Values to the right in the lower panels represent the number of molecules associated with the respective pathways. (**F** and **G**) Manual functional annotation and comparison of genes identified to be differentially expressed following imatinib-pretreatment at 3 hpi (**F**) and 24 hpi (**G**) with the Harmonizome database. The bars represent the number of differentially expressed genes in our respective datasets that associate with the metabolism, inflammation and fibrosis harmonizome dataset, respectively. Inset **F**: Expression of the identified fibrosis- and inflammation-associated genes in cells of the unchallenged NVU (mouse brain vasculature atlas from Vanlandewijk *et al.* 2018 (11)). EC, endothelial cells; PC, pericytes; SMC, vascular smooth muscle cells; AC, perivascular astrocytes; FB, fibroblast-like cells; Non NVU, not expressed in the unchallenged NVU. (**H** and **I**) Comparison of imatinib regulated genes at 3 hpi (**H**) and 24 hpi (**I**) with transcriptome analysis of the perihematomal area of stroke patients reveals high overlap, especially with fibrosis- and inflammation-associated genes. Data points represent individual animals and bars the group mean ± SEM. The dashed

line shows the sham-operated group mean. Two-way ANOVA with Fisher's LSD test (**B** and **C**); Right-tailed Fisher's exact test (**D** and **E**). Statistical significance: ns, non-significant; * $P < 0.05$; ** $P < 0.01$.

Supplementary Fig. S3. Protzmann et al.

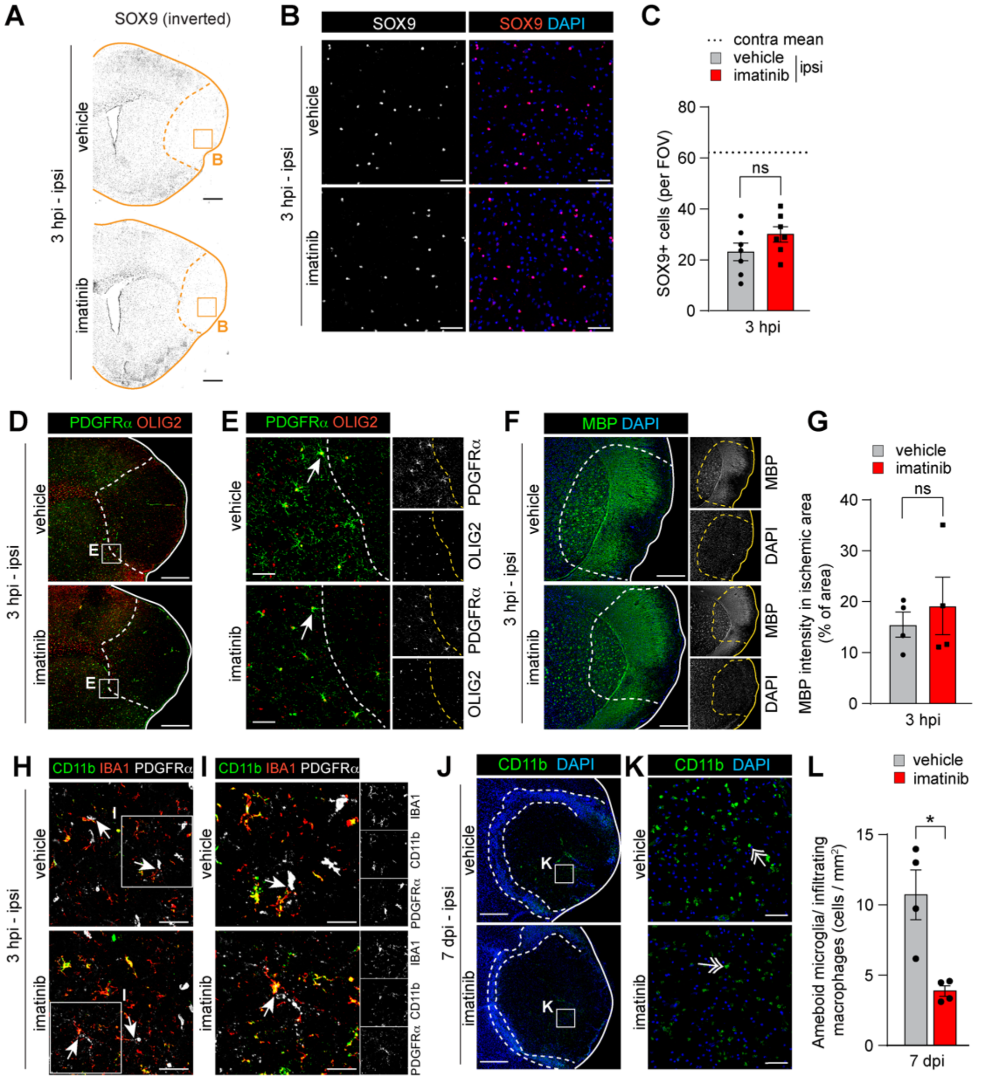

Supplementary Figure 3. Imatinib does not affect astrocyte number, myelination or microglia activation in the acute phase post MCAO but inhibits microglia activation/macrophage infiltration in the early chronic phase. Representative images of immunofluorescent stainings and quantification in brain sections from vehicle and imatinib pretreated mice collected at 3 hpi (**A-I**) and 7 dpi (**J-L**). Ipsilateral overviews (**A**) and high magnification images acquired in the ischemic area (**B**) from stainings for SOX9. (**C**) Quantification of SOX9$^+$ nuclei in the ischemic area (*N* = 7). Ipsilateral overviews (**D**) and high magnification images acquired in the ischemic border (**E**) from co-stainings for PDGFRα and OLIG2. Arrows, PDGFRα$^+$;OLIG2$^+$ cells. (**F**) Ipsilateral overviews from co-stainings for myelin basic protein (MBP). (**G**) Quantification of MBP staining intensity above a set threshold in the ischemic area (*N* = 4). High magnification images (**H**) and blow-ups (**I**) acquired in the ischemic border from co-stainings for IBA1, CD11b and PDGFRα. Ipsilateral overviews (**J**) and high magnification images (**K**) acquired in the ischemic core from stainings for CD11b. Two-headed arrow, CD11b$^+$ ameboid cells. (**L**) Quantification of ameboid (activated) microglia/ infiltrating macrophages in the ischemic core (*N* = 4). Single confocal planes (**D**, **F** and **J**), maximum intensity projections of confocal z-stacks (**A**, **B**, **E**, **H**, **I** and **K**). Ischemic area (**A-F**) and fibrotic rim (**H**) outlined with dashed lines. Data points represent individual animals, bars the group mean ± SEM and the dashed line **C** the contralateral group mean. Two-tailed, unpaired t-test with Welch's correction (**C**, **G** and **L**). Statistical significance: ns, non-significant; * $P < 0.05$. Scale bars: 500 µm (**A**, **D**, **F** and **J**); 50 µm (**B**, **E**, **H** and **K**); 25 µm (**I**).

Supplementary Fig. S4. Protzmann et al.

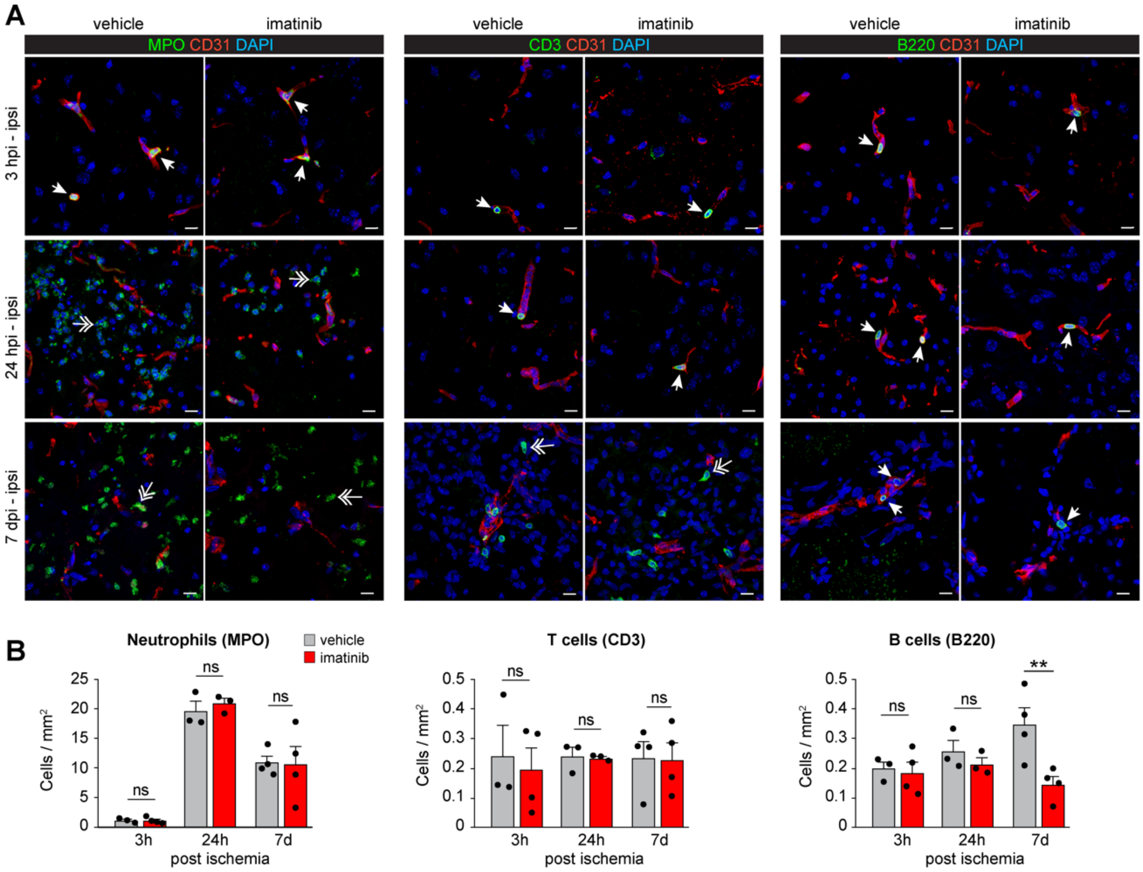

Supplementary Figure S4. Imatinib appears to have limited effect on neutrophil, T- and B-cell infiltration after MCAO. (**A**) Immunofluorescent stainings in brain sections from vehicle and imatinib pretreated mice collected at 3 hpi, 24 hpi and 7 dpi. Representative maximum intensity projections of confocal z-stacks from stainings for MPO (neutrophils), CD3 (T cells) or B220 (B cells) are shown. Vessels visualized with CD31. Arrows, immune cells in the blood vessel lumen; Two-headed arrows, immune cells transmigrated into the brain parenchyma. (**B**) Quantification of MPO^+, $CD3^+$ and $B220^+$ cells in the brain parenchyma. n=3-4 animals per time and treatment. Data points represent individual animals and bars the group mean ± SEM. Two-way ANOVA with Fisher's LSD test (**B**). Statistical significance: ns, non-significant; ** *P* < 0.01. Scale bars, 10 µm (**A**).

Supplementary Fig. S5. Protzmann et al.

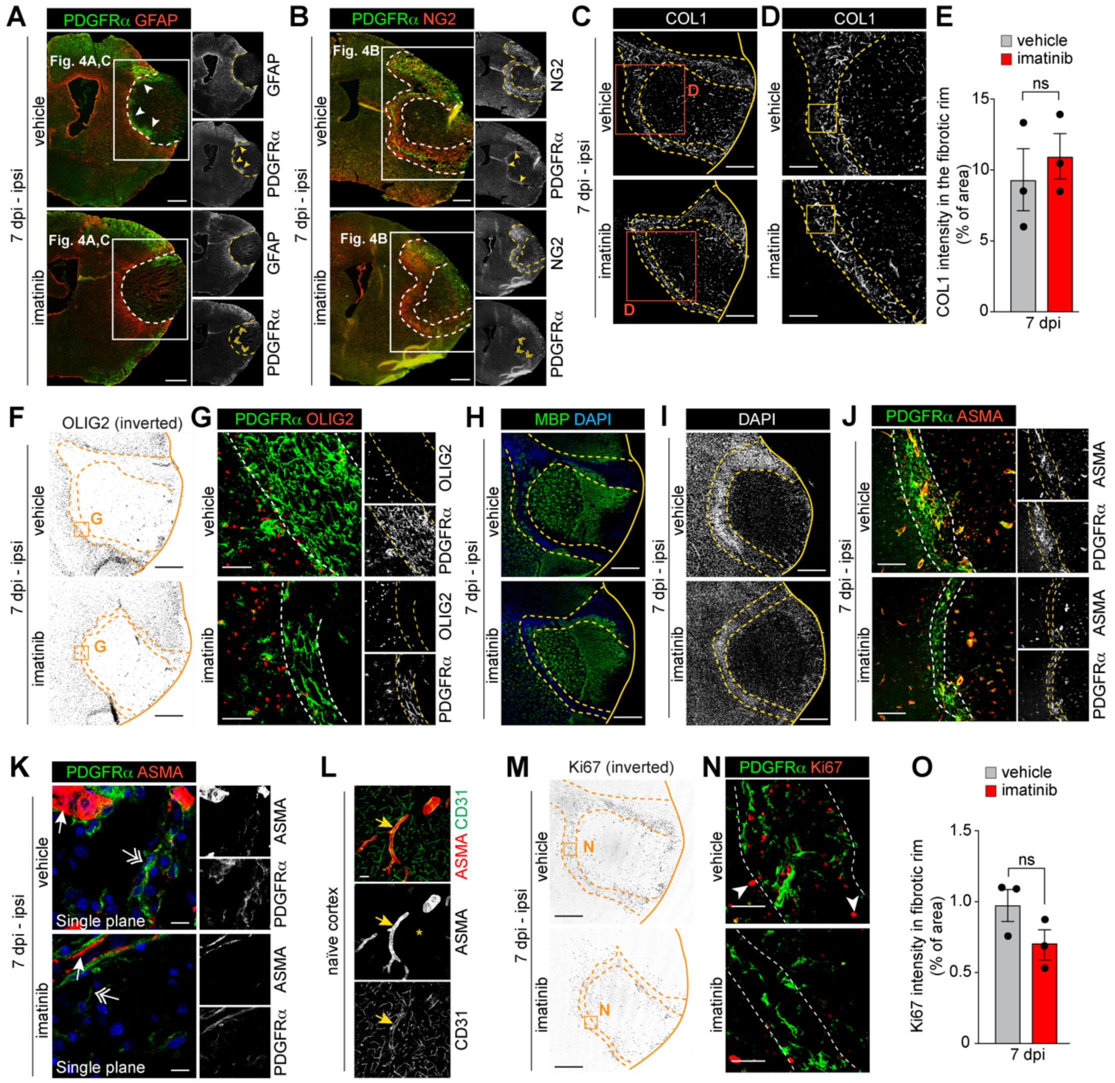

Supplementary Figure 5. Imatinib attenuates MCAO-induced myofibroblast expansion in the early chronic phase after ischemia. (**A** and **B**) Ipsilateral overviews from stainings for GFAP (**A**) and NG2 (**B**). (**C** and **D**) Ipsilateral overviews (**C**) and high magnification images acquired in the ischemic border (**D**) of stainings for collagen1 (COL1). (**E**) Quantification of COL1 expression in the fibrotic rim (demarcated in **D**) ($N = 3$). (**F** and **G**) Ipsilateral overviews (**F**) and high magnification images acquired in the fibrotic rim (**G**) from staining for PDGFRα and OLIG2. (**H**) Ipsilateral overviews from stainings for MBP. (**I**) Ipsilateral overviews of the highly nucleated area in the rim of the fibrotic lesion as detected by DAPI. (**J**) High magnification images acquired in the fibrotic rim from co-stainings for PDGFRα and ASMA. (**K**) Single plane confocal image from co-stainings for PDGFRα and ASMA (maximum intensity projection shown in **Figure 4M**). Two-headed arrows, PDGFRα^{+};ASMA^{+} parenchymal cells; Arrows, ASMA^{+} vSMCs. (**L**) High magnification images from co-staining for ASMA and CD31 in the naïve cortex. Arrow, ASMA expression is restricted to vSMCs in medium-to-large vessels; Asterisk, no ASMA expression is found along microvessels or in the brain parenchyma. (**M** and **N**) Ipsilateral overviews (**M**) and high magnification images (**N**) of co-stainings for Ki67 and PDGFRα. Arrowheads, proliferating cells negative for PDGFRα immunoreactivity. (**O**) Quantification of Ki67 expression in the fibrotic rim (demarcated in **M**) ($N = 3$). Representative images of immunofluorescent stainings in brain sections from vehicle and imatinib pretreated mice collected at 7 dpi (**A-K**, **M** and **N**) or from naïve uninjured mice (**L**). Stitched epifluorescent tiles (**A** and **B**), single confocal planes (**C-F**, **H**, **I** and **M**), maximum intensity projections of confocal z-stacks (**G**, **J-L** and **N**). Dashed lines demarcate the glia border/scar (**A** and **B**) and the myofibroblast scar in the fibrotic rim (**C-K**). Data points represent individual animals, bars the group mean ± SEM. Two-tailed, unpaired t-test with Welch's correction (**E** and **O**). Statistical significance: ns, non-significant. Scale bars: 500 µm (**A-C**, **F**, **H**, **I** and **M**); 250 µm (**D** and **J**); 50 µm (**G**, **L** and **N**); 10 µm (**K**).

Supplementary Fig. S6. Protzmann et al.

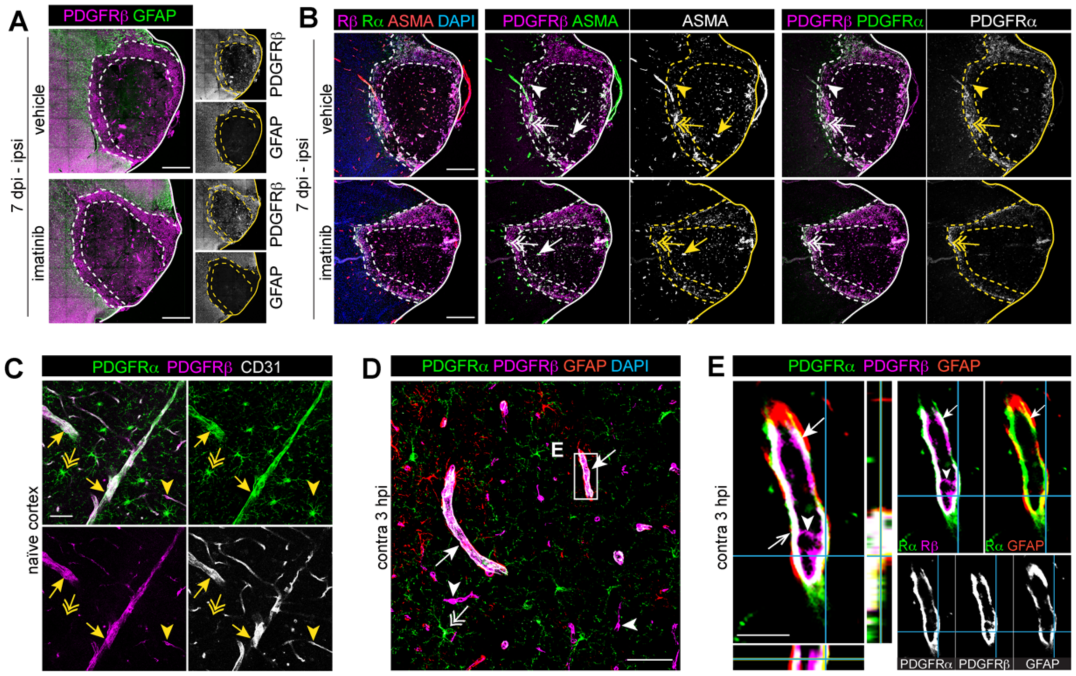

Supplementary Figure 6. The PDGFRβ⁺ fibrotic scar appears to be unaffected by imatinib treatment after MCAO. (**A**) Ipsilateral overviews from co-stainings for PDGFRβ and GFAP. (**B**) Ipsilateral overviews from co-staining for PDGFRβ (Rβ), PDGFRα (Rα) and ASMA at 7 dpi (single channel images of PDGFRβ shown in **Figure 5A**). Two-headed arrows, PDGFRα^{high};PDGFRβ^{high} cells. Arrowheads, PDGFRα^{low};PDGFRβ^{high} cells. Arrows, ASMA^{+};PDGFRβ^{+} vSMCs. (**C**) High magnification images from co-stainings for PDGFRα and PDGFRβ in the naïve cortex. Vessels visualized with CD31. Arrows, perivascular PDGFRα^{+};PDGFRβ^{+} cells around medium-to-large sized vessels. Two-headed arrows, non-vascular parenchymal PDGFRα^{+} glia cells (NG2-glia/OPCs). Arrowheads, PDGFRβ^{+} pericytes along capillaries. (**D**) High magnification image from co-staining for PDGFRα, PDGFRβ and GFAP acquired in the contralateral hemisphere. Arrows, PDGFRα^{+};PDGFRβ^{+};GFAP^{+} perivascular cells around medium-to-large sized vessels. Two-headed arrows, non-vascular parenchymal PDGFRα^{+} glia cells (NG2-glia/OPCs). Arrowheads, PDGFRβ^{+} pericytes along capillaries. (**E**) Single plane confocal image (**box in D**) and orthogonal projection from a co-staining for PDGFRα, PDGFRβ and GFAP. Arrows, PDGFRα^{+};PDGFRβ^{+};GFAP^{+} perivascular cells. Arrowhead, PDGFRβ^{+} vSMC. Representative images of immunofluorescent stainings in brain sections from vehicle and imatinib pretreated mice collected at 7 dpi (**A** and **B**), in the naïve (**C**) or contralateral cortex (**D** and **E**). Single plane confocal images (**A** and **B**), maximum intensity projections of confocal z-stacks (**C-E**). Dashed lines demarcate the PDGFRβ^{high} scar in the fibrotic rim (**A** and **B**). Scale bars: 500 µm (**A** and **B**); 50 µm (**C** and **D**); 10 µm (**E**).

Supplementary Fig. S7. Protzmann et al.

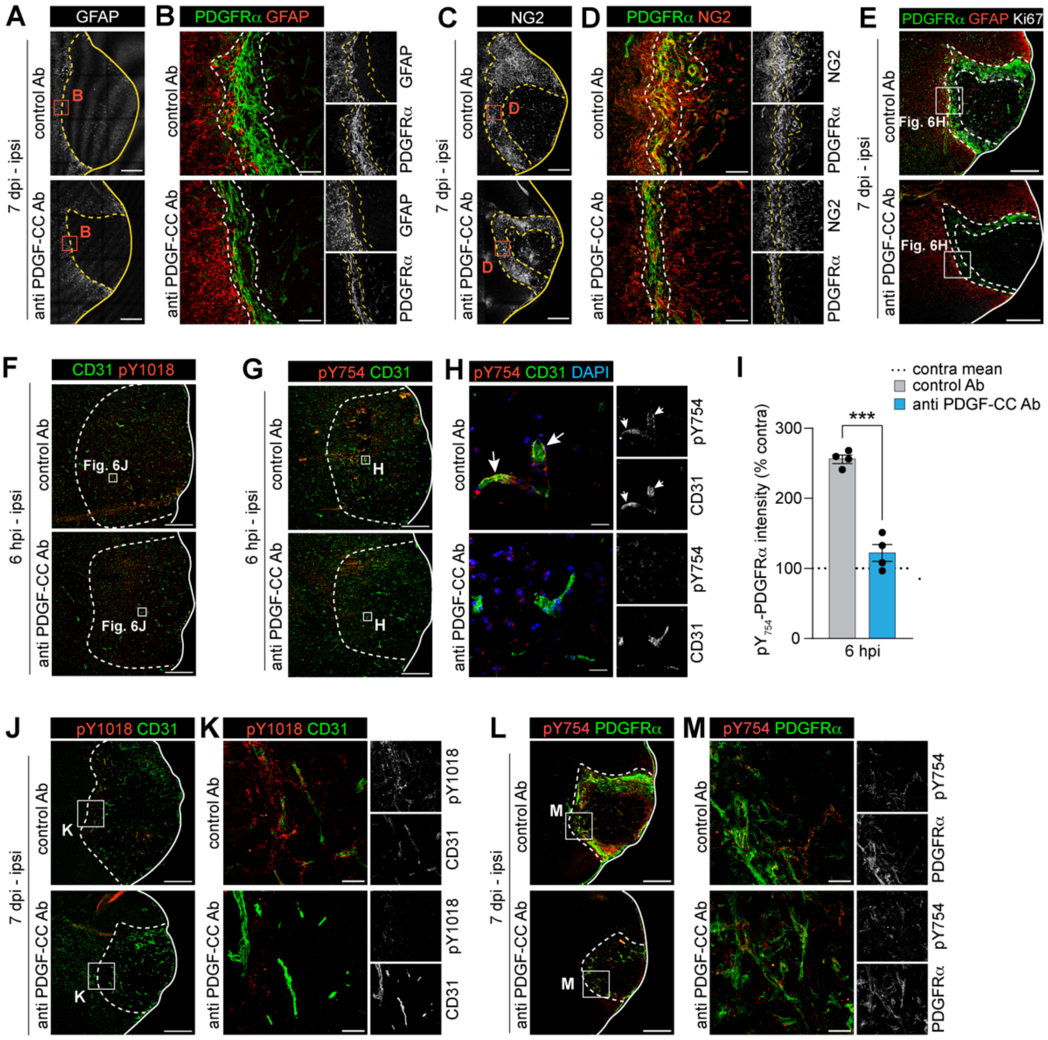

Supplementary Figure 7. Anti-PDGF-CC antibody treatment inhibits MCAO-induced activation of PDGFRα and myofibroblast expansion in the fibrotic rim. (**A** and **B**) Ipsilateral overviews (**A**) and high magnification images (**B**) from co-stainings for PDGFRα and GFAP. (**C** and **D**) Ipsilateral overviews (**C**) and high magnifications images (**D**) from co-stainings for PDGFRα and NG2. (**E**) Ipsilateral overviews from co-stainings for PDGFRα, GFAP and Ki67. (**F**) Ipsilateral overviews from co-stainings for phospho-PDGFRα (pY1018) and CD31 at 6 hpi. (**G** and **H**) Ipsilateral overviews (**G**) and high-magnifications (**H**) from co-stainings for phospho-PDGFRα (pY754) and CD31 at 6 hpi. Arrows, phosphorylation of perivascular PDGFRα. (**I**) Quantification of perivascular phospho-PDGFRα (pY754) intensity in the ischemic area normalized to the contralateral hemisphere ($N = 4$). (**J** and **K**) Ipsilateral overviews (**J**) and high magnification images acquired in the fibrotic rim (**K**) from stainings for phospho-PDGFRα (pY1018) and CD31 at 7 dpi. (**L** and **M**) Ipsilateral overviews (**L**) and high magnifications (**M**) from stainings for total PDGFRα and phospho-PDGFRα (pY754). Representative images of immunofluorescent stainings in brain sections and quantifications from control and anti-PDGF-CC antibody treated mice at 6 hpi (**F-I**) and 7 dpi (**A-E** and **J-L**). Singe plane confocal images (**A-G**, **J** and **L**), maximum intensity projections of confocal z-stacks (**H**, **K** and **M**). Dashed lines demarcate the glia scar/border (**A**, **C**, **J** and **L**), the PDGFRα^{+} myofibroblast scar in the fibrotic rim (**B**, **D** and **E**) and the ischemic border (**F** and **G**). Data points represent individual animals and bars the group mean ± SEM. The dashed line in **E** shows the contralateral group mean. Two-tailed, unpaired t-test with Welch's correction (**E**). Statistical significance: *** $P < 0.001$. Scale bars: 500 µm (**A**, **C**, **E-G**, **J** and **L**); 100 µm (**B** and **C**); 50 µm (**K** and **M**); 20 µm (**H**).

Supplementary Fig. S8. Protzmann et al.

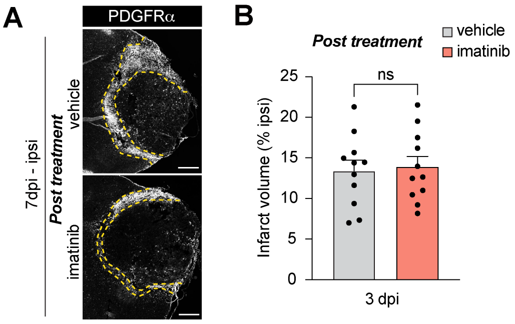

Supplementary Figure 8. Imatinib post-treatment reduces myofibroblast expansion in the early chronic phase post MCAO but has no effect on infarct size in the subacute phase. (**A**) Ipsilateral overview from stainings for PDGFRα at 7 dpi in vehicle or imatinib *post-treated* mice. Dashed lines demarcate the PDGFRα^{+} myofibroblast scar in the fibrotic rim. (**B**) Quantification of lesion volume at 3 dpi in mice *post-treated* with imatinib or vehicle. n=11 animals per treatment. Data points represent individual animals and bars the group mean ± SEM. Two-tailed, unpaired t-test with Welch's correction. Statistical significance: ns, non-significant. Scale bars: 500 µm (**A**).

Supplementary Video S1. Lateralized sensory-motor integration test after MCAO.

Example of lateralized sensory-motor integration using the corridor task in a sham operated mouse and in vehicle- and imatinib-pretreated mice 7 days after MCAO. Sham mice explore sugar pellets on both sides of the corridor, while vehicle treated mice displayed a contralateral neglect exploring pellets primarily on the ipsilateral side of the ischemic infarct. Imatinib treatment alleviated the contralateral neglect/ ipsilateral bias.

Supplementary table 1.

Differentially expressed genes 3 hours post MCAO (down or upregulated in imatinib samples). This table includes a list of differentially expressed genes from vascular fragments of vehicle compared to imatinib-treated mice 3 hours post MCAO. Genes with P-value < 0.05 and a log2 (0.5) fold change are shown. For each gene the association with fibrosis, inflammation or metabolism is indicated.

Supplementary table 2.

Differentially expressed genes 24 hours post MCAO (down or upregulated in imatinib samples). This table includes a list of differentially expressed genes from vascular fragments of vehicle compared to imatinib-treated mice 24 hours post MCAO. Genes with P-value < 0.05 and a log2 (0.5) fold change are shown. For each gene the association with fibrosis, inflammation or metabolism is indicated.

Supplementary table 3.

Comparison 3h post MCAO to human data. Transcripts that were significantly regulated in vascular fragments isolated from imatinib-treated mice compared to vehicle controls at 3 hours post MCAO were compared to microarray data from human perihematomal area (GSE24265). 49% of the common genes for both data were differentially expressed in both human and our data set. For each gene the association with fibrosis, inflammation or metabolism is indicated.

Supplementary table 4.

Comparison 24h post MCAO to human data. Transcripts that were significantly regulated in vascular fragments isolated from imatinib-treated mice compared to vehicle controls at 24 hours post MCAO were compared to microarray data from human perihematomal area (GSE24265). 48% of the common genes for both data were differentially expressed in both human and our data set. For each gene the association with fibrosis, inflammation or metabolism is indicated.
